# Targeted Theranostic Nanoparticles for Brain Tumor Treatment

**DOI:** 10.3390/pharmaceutics10040181

**Published:** 2018-10-09

**Authors:** Maria Mendes, João José Sousa, Alberto Pais, Carla Vitorino

**Affiliations:** 1 Faculty of Pharmacy, University of Coimbra, 3000-548 Coimbra, Portugal; mariamendes1093@gmail.com (M.M.); jjsousa@ff.uc.pt (J.J.S.); 2 Center for Neurosciences and Cell Biology (CNC), University of Coimbra, 3004-504 Coimbra, Portugal; 3 LAQV, REQUIMTE, Group of Pharmaceutical Technology, 3000-548 Coimbra, Portugal; 4 Coimbra Chemistry Centre, Department of Chemistry, University of Coimbra, 3004-535 Coimbra, Portugal; pais@qui.uc.pt

**Keywords:** nanotechnology, glioblastoma, theranostics, gold nanoparticles, lipid nanoparticles, active targeting

## Abstract

The poor prognosis and rapid recurrence of glioblastoma (GB) are associated to its fast-growing process and invasive nature, which make difficult the complete removal of the cancer infiltrated tissues. Additionally, GB heterogeneity within and between patients demands a patient-focused method of treatment. Thus, the implementation of nanotechnology is an attractive approach considering all anatomic issues of GB, since it will potentially improve brain drug distribution, due to the interaction between the blood–brain barrier and nanoparticles (NPs). In recent years, theranostic techniques have also been proposed and regarded as promising. NPs are advantageous for this application, due to their respective size, easy surface modification and versatility to integrate multiple functional components in one system. The design of nanoparticles focused on therapeutic and diagnostic applications has increased exponentially for the treatment of cancer. This dual approach helps to understand the location of the tumor tissue, the biodistribution of nanoparticles, the progress and efficacy of the treatment, and is highly useful for personalized medicine-based therapeutic interventions. To improve theranostic approaches, different active strategies can be used to modulate the surface of the nanotheranostic particle, including surface markers, proteins, drugs or genes, and take advantage of the characteristics of the microenvironment using stimuli responsive triggers. This review focuses on the different strategies to improve the GB treatment, describing some cell surface markers and their ligands, and reports some strategies, and their efficacy, used in the current research.

## 1. Introduction

Drug delivery to the brain is a major challenge, due to the high brain protection against the entry of foreign molecules. Many molecules are described having action in the brain disorders that fail clinical testing, due to their inability to cross the blood–brain barrier (BBB). This imposes the need for developing more effective delivery strategies. Nanostructured delivery systems (NDS) are complex nanocarriers that have sparked particular interest in biological applications, such as therapy and diagnosis, in some cases as multifunctional nanosystems, claiming two applications at the same time. NDS can be structurally divided into two parts: an external layer (shell), able to be functionalized with a variety of small molecules, proteins, metal ions, and/or polymers, and an internal layer (core), which is essentially the central portion of the NPs and chemically composed of different materials or a simple reservoir (comprising drugs and contrast agents) as in the case of liposomes. They have the ability to carry various therapeutic molecules, including small molecules, proteins, peptides and genetic material. In this context, theranostic nanoparticles arise as promising systems bringing new opportunities to overcome the restrictions of current brain tumor treatment/diagnosis options in the clinic, protecting the drug from metabolism, conveying two or more drugs simultaneously with synergistic effect, enabling a controlled and specific release of the drug, thereby decreasing the respective side effects.

In this review, the brain tumor classification and biological features are described, followed by the obstacles imposed to an effective treatment. Subsequently, recent advances and clinical applications of nanoparticles (NPs) in brain tumor therapeutics are addressed, with focus on: (i) the several approaches for brain targeting; (ii) the combination of both imaging and therapeutic functions (i.e., theranostics); and (iii) the use of NDS for glioblastoma treatment, including current research, and pre-clinical and clinical investigation. The importance of nanotheranostics in a personalized medicine perspective and the difficulties found for the respective clinic translation are also considered.

## 2. Glioblastoma

Brain tumors are a heterogeneous group of primary and metastatic neoplasms in the central nervous system (CNS), characterized by poor prognosis and patient low survival rate. They are classified by the World Health Organization (WHO) according to a grade of malignancy that is closely related to diagnosis, varying from grade I, which is characterized by lesions with low proliferative potential and possibility of cure, to grade IV, which is described as cytological malignant, mitotically active neoplasms that are typically associated with extensive invasion of the surrounding healthy tissue and rapid proliferation linked to disease evolution (Table 1) [1]. Glioblastoma (GB), a grade IV glioma, is the most frequent primary brain tumor, the most aggressive and lethal in humans, with a patient survival rate between 8 and 14 months after diagnosis [2]. The poor prognosis is partly because of the multidrug resistance, limited surgical resection, critical importance of the residual glioma cells that have capacity to develop a new primary tumor, and surgery-dependent malignance. One of the most challenging problems in GB therapy is its particularly complex and heterogeneous molecular biology. Consequently, the response to the same treatment by different patients results in equally different prognosis. Current management of GB frequently consists of surgical resection, followed by radiotherapy (RT) and adjuvant chemotherapy, both treatments inducing DNA damage (Table 1) [3]. The low therapeutic efficacy, associated to a large side effects spectrum, involving damage in healthy tissues, require regular invasive dose regimens. Generally, the long-term patient quality of life is poor, because the outlying tissues must be subjected to treatment, otherwise the tumor will reappear, which make the treatment of GB even more difficult. The development of GB still varies according to sex, being more prevalence in males, age (>45 years) and existence of genetic disorders [4].

In the next section, the barriers to GB treatment, particularly, BBB and BBTB, are reviewed, as well as the emerging advances in the treatment of GB using NPs as a promising strategy, with emphasis on drug delivery, targeting and diagnosis in real-time.

### 2.1. Barriers and Transport Pathways for the Treatment of Glioblastoma

Several obstacles limit GB treatment efficacy, including the structural complexity of the brain, the blood–brain barrier (BBB) and blood–brain–tumor barrier (BBTB), the heterogeneous and invasive nature of the tumor, insufficient accumulation of drugs at the site of the tumor and resistance of chemotherapeutics.

#### 2.1.1. Blood–Brain Barrier

**Figure 1:**
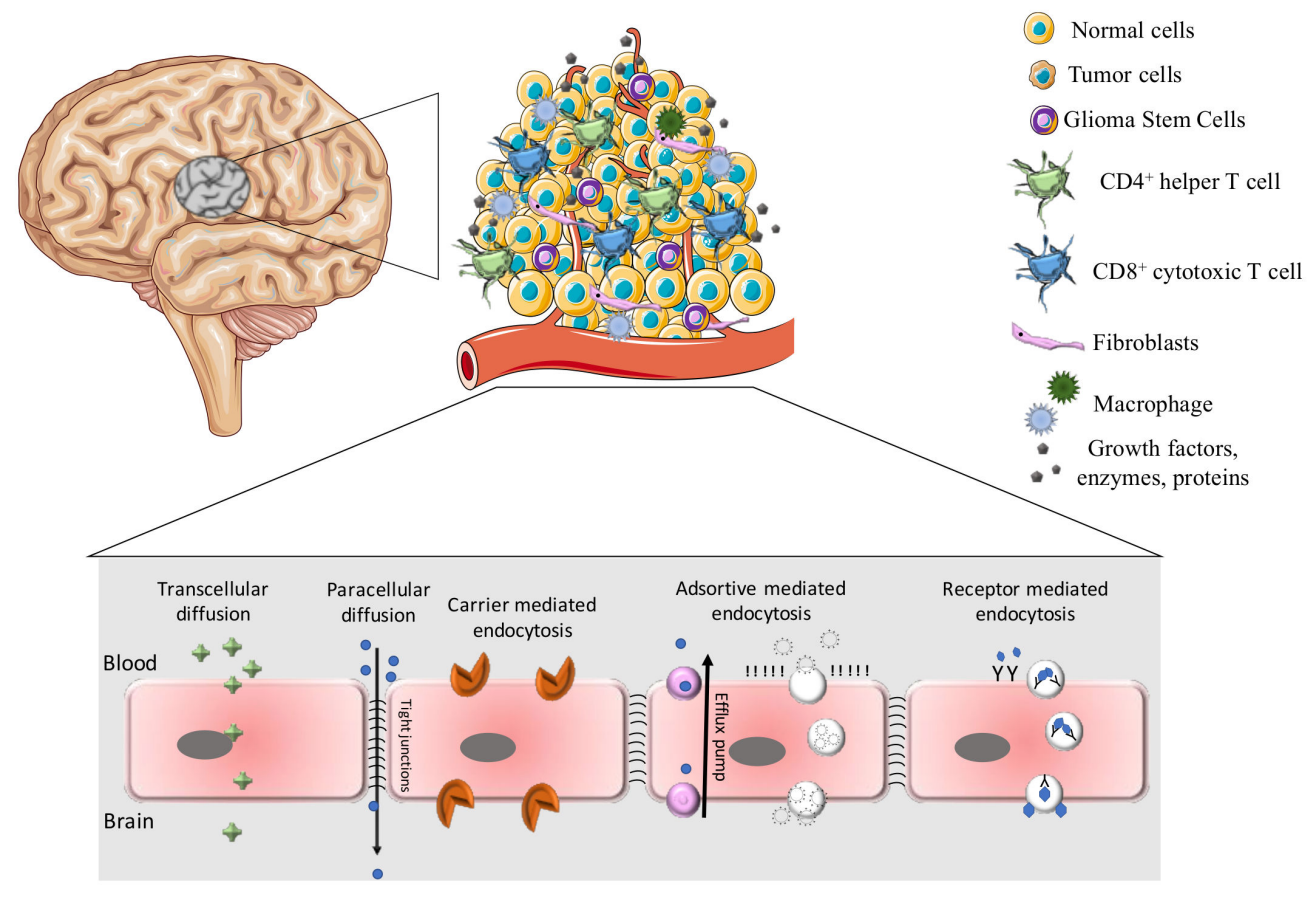
Brain tumor structure and therapeutic brain delivery strategies for targeting the physiological blood–brain barrier (BBB), including transcellular lipophilic diffusion, paracellular hydrophobic diffusion, carrier mediated endocytosis, ATP-binding cassette (ABC)-transporters, adsorptive mediated transcytosis and receptor mediated endocytosis. In addition, it is represented the tumor microenvironment with tumor cells, glioma stem cells, CD4^+^ helper T cells, CD8^+^ cytotoxic T cells, fibroblasts, macrophages, growth factors, enzymes and proteins.

The BBB severely restricts drug transport into the brain by serving as a physical (tight junctions), metabolic (enzymes) and immunological barrier [18]. The BBB is responsible for regulating the ionic composition for synaptic signaling function and providing brain nutrients, which prevents the entry of any macromolecules and protects the CNS from neurotoxic substances [18]. The anatomical structure of the BBB consists of a monolayer of non-fenestrated blood vessel endothelial cells attached by tight junctions (TJs) through the interaction of cell adhesion molecules, pericytes, and astrocytes, which provides a structural support by holding the cells together [19]. In addition, the barriers created by TJs among cerebral endothelial cells (ECs), the choroid plexus epithelial cells and the cells of the arachnoid epithelium prevent the access through the paracellular pathway [20,21]. The BBB microenvironment is constituted by macrophages, fibroblasts, neuronal cells, basal membranes and microglia [22]. The presence of numerous enzymes in cerebral ECs and efflux transport mechanisms, e.g., P-glycoprotein (P-gp), constitute major obstacles for molecules to cross the BBB. Several BBB transport pathways are described depending on physicochemical properties of drug molecules, such as paracellular aqueous pathways, transcellular lipophilic pathways, transport proteins, receptor-mediated transcytosis and adsorptive transcytosis (Figure 1). Passive diffusion depends on molecular weight and lipophilicity. Additionally, the capacity of molecules to form hydrogen bonds will limit their diffusion through the BBB. Only a few small molecule drugs cross the BBB by lipid-mediated free diffusion, unless the drug possesses a molecular weight of less than 400 Da and forms less than 8 hydrogen bonds [23,24]. The difficulty of crossing the BBB is even more evident in the case of large molecule drugs. About 100% of large molecule drugs do not pass the BBB, including proteins and enzymes, monoclonal antibodies, or gene therapy. Brain diffusion of exogenous molecules is limited by ATP-binding cassette (ABC) transporters, efflux transport proteins placed at the luminal endothelial cell membrane. Alternative pathways traversing the BBB have been investigated for their potential application in invasive drug delivery. In addition to the transcellular lipophilic pathway for small, lipophilic molecules, the other transport routes include adsorptive-mediated (AMT), carrier-mediated (CMT), and receptor-mediated transcytosis (RMT) have been employed for targeting the brain [25,26,27].

##### Adsorptive-Mediated Transport

Adsorptive-mediated transport (AMT) is a nonspecific process by which macromolecules are transported within membrane bound vesicles between apical and basolateral domains of polarized cells. This process is possible due to the abundance of polyanions surrounding the BBB endothelial cells that are able to interact electrostatically with circulating cationic molecules. This activity does not involve particular membrane receptors. The electrostatic interaction between positively-charged molecules and negative charge of BBB cells has been used to favor GB targeting. Cationic albumin-conjugated (CBSA), gemini surfactants, charged-cell penetrating peptides (CPP) or cationized immunoglobulin G are some examples of positive molecules that are strategically coupled to the surface of NDS [28,29,30,31,32,33,34,35]. However, electrostatic interactions at the BBB are not always consistent [36]. Surface charge can influence the interaction between nanoparticle and cells, affecting in particular processes such as cellular uptake, biodistribution, metabolism and excretion. Some works have demonstrated that positive-NPs are more easily internalized than neutral- and negative-NPs. However, positive-NPs have more tendency to adsorbed serum proteins and other biomolecules in circulation and consequently a new interface is formed, also known as corona [37,38,39,40]. These somewhat altered NPs display in vivo a behavior different from that of the original NPs, impacting upon the specific targeting between ligands and receptor [41]. Thus, the corona formation needs to be further studied, and directed to the biological application [42]. In addition, it should be taken into consideration that the original charge of the nanoparticle is likely to affect the characteristics of corona and this must be kept in mind when designing NPs, especially in case of brain delivery.

##### Carrier-Mediated Transcytosis

Carrier-mediated transcytosis (CMT) is substrate-selective and the transport rate is dependent on the degree of occupation of the carrier. CMT enables spontaneous internalization of small biomolecules, including nutrients (glucose), hormones, amino acids, bile salts, and monocarboxylic acids by passive diffusion. This type of transport is used for the delivery of nutrients, such as glucose, amino acids, and purine bases, to the brain. Eight different nutrient transport systems have been recognized, each one transporting a group of nutrients of the same structure [43]. Glucose is essential for brain function and crosses the BBB via a specific transporter by glucose receptors (GLUT). Beyond the nutrients, there are several amino acid transporters at the BBB to maintain brain homeostasis, including neutral amino acids transporter (NAAT), cationic amino acids transporter (CAATs), anionic amino acids transporters (AAATs) and beta amino acid transporter (βAATs) [44]. The choline transporter (ChT) is used for transportation of choline and thiamine to support the neurological supplies of brain [45,46]. Peptide (PT), fatty acid (FAT) and nucleoside transporters (NTs) are also expressed in the BBB (Table 2). Therefore, only drugs that closely mimic the endogenous carrier substrates will be taken up and transported into the brain. Nutrients and hormones or carrier systems, at the BBB level, have been exploited for brain delivery, increasing metabolic stability, reduced clearance, and improved BBB transport.

##### Receptor-Mediated Transcytosis

Receptor mediated transcytosis (RMT) is characterized by high specificity, selectivity and affinity and considered one of the best strategies for brain targeted drug delivery [60,61]. The mechanisms occur when the ligand binds to a transmembrane receptor, which is expressed on the apical plasma membrane of the endothelial cell (Figure 1). The transferrin receptor (TfR), the low density lipoprotein receptor (LDLR), the insulin receptor (IR) and the nicotinic acetylcholine receptors (nAChRs) are examples of receptor expression on the BBB, and could be targeted by ligands, such as endo- or exogenous ligands (Table 3). The TfR is one of the most characterized RMT for brain targeting, due to their high expression on endothelial cells on the brain capillary endothelial cells (BCECs) and tumor cells. Different transferrin-NDS for drug delivery through the BBB have been developed, and in vitro and in vivo brain-targeting efficiencies have been reported [62,63]. However, transferrin is a natural ligand, and consequently, TfR is saturated for the endogenous TF, having a natural competition between endogenous TF and the TF-NPs. For this reason, OX26 and RI7217 have been studied in order to avoid this competition [64,65,66]. Both are monoclonal antibodies with a high affinity for TfR. LDLR, expressed on the BCECs, have endogenous ligands including cholesterol and apolipoprotein (Apo) [67,68,69,70,71,72]. Angiopep-2, apolipoprotein B or E (ApoB or ApoE), polysorbate 80 (PS80) and, more recently, polyoxyethylene sorbitol oleate (PSO) have been described as exogenous ligands [73,74,75,76,77,78]. Insulin receptor (IR) is a transmembrane glycosylated protein sensible to the transport of blood-borne insulin into the brain [79,80]. Nicotinic acetylcholine receptors (nAChRs), highly expressed on the capillary endothelium of the brain, have been used to facilitate BBB crossing of NDS. The large expression of nAChRs in the brain and their susceptibility to the inhibition by peptide neurotoxins provide them the capacity to mediate peptide-based delivery of various therapeutic agents to the brain [81]. ABC (ATP-binding cassette) transporters are ATP-driven drug efflux pumps expressed in the BBB which embrace P-glycoprotein (P-gp) and members of the multidrug resistance related proteins (MDRs, especially MRP1, MRP2, MRP4 and MRP5), and breast cancer resistance protein (BCRP and ABCG2) [82,83]. These proteins are expressed at the luminal side of brain capillaries and are a barrier for the brain uptake of a huge diversity of lipophilic molecules, potentially toxic metabolites, xenobiotics, and drugs [84]. Even though different RMTs are described in the literature as possible active targets for the brain, they are also present in other biological barriers (e.g., intestinal membrane and liver) [85,86,87]. For this reason, this is an ineffective method for active targeting NPs. Thus, active targeting strategy needs to be well designed to avoid off-target NPs accumulation in other tissues.

**Figure 2:**
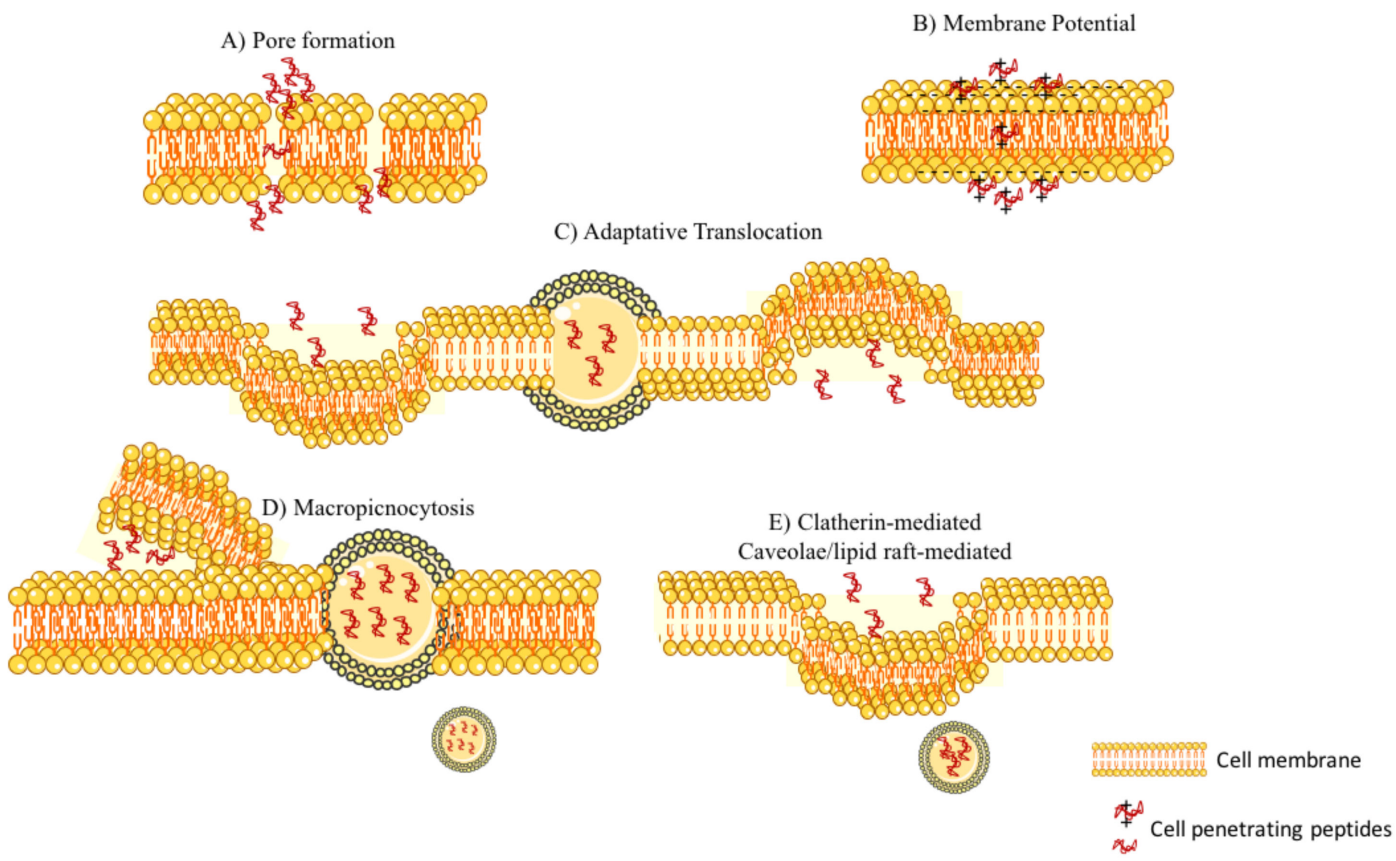
Intracellular pathways of cell entry for charged-cell penetrating peptides (CPPs): (**A**) Transient toroid pore formation; (**B**) membrane potential; (**C**) adaptative translocation; (**D**) micropinocytosis; and (**E**) clathrin-mediated endocytosis and caveolae/lipid raft-mediated endocytosis.

Cell penetrating peptides (CPP), that show great capacity in BBB transport, have the ability to transport protein or peptides into cells in a nonspecific, receptor-independent manner. CPPs are small peptides with appealing capability of membrane translocation and cellular uptake. Moreover, CPPs have the advantage of being non-immunogenic when compared with antibodies, which make them interesting as targeting strategy for a broad range of therapeutics [88,89,90,91]. They can vehicle the molecules that are attached to them across the cell membrane, into the cytoplasm and to the nucleus, associated either through physical or covalent interactions. According to their physicochemical properties, CPPs can be grouped into different classes, such as cationic, hydrophobic, and amphipathic [92]. Some CPPs, named as activable cell-penetrating peptides (ACPPs), can be synthetized to be active under the action of certain triggers, when reached the target site, such as pH, proteinase, and UV light (Table 3) [93,94,95,96,97,98]. ACPPs are promising methods due to their stealth properties. Among all these triggers, enzyme-triggered systems have been widely studied and applied in tumor imaging and therapy. Recently, chemotherapeutic NDS decorated with CPPs for tumor treatment have been developed and assessed, including the GB [99,100,101]. They can intermediate the cellular uptake of a wide range of macromolecules and thus have called significant attention as drug delivery in vivo strategy. Different CPPs have been described, and their classification consider multiple approaches, such as source, function, sequence, mechanism of uptake, and biomedical application (Figure 2 and Table 1).

Another approach described in the literature is the conjugation of tumor targeting peptides (TTP) with CPP [102,103,104,105]. TTP are molecules that interact particularly with overexpressed receptors on tumor cells (cell markers are represented in Table 3). However, they may not be able to reach the target on their own. For this reason, the interaction between TTP and CPP has been investigated, used as a synergistic effect, facilitating the translocation of the conjugate unit to target tumor sites with enhanced specificity and selectivity [91]. Several experimental studies show that TTPs alone have considerably reduced easiness to translocate therapeutic molecules.

#### 2.1.2. Blood–Brain–Tumor Barrier

**Figure 3:**
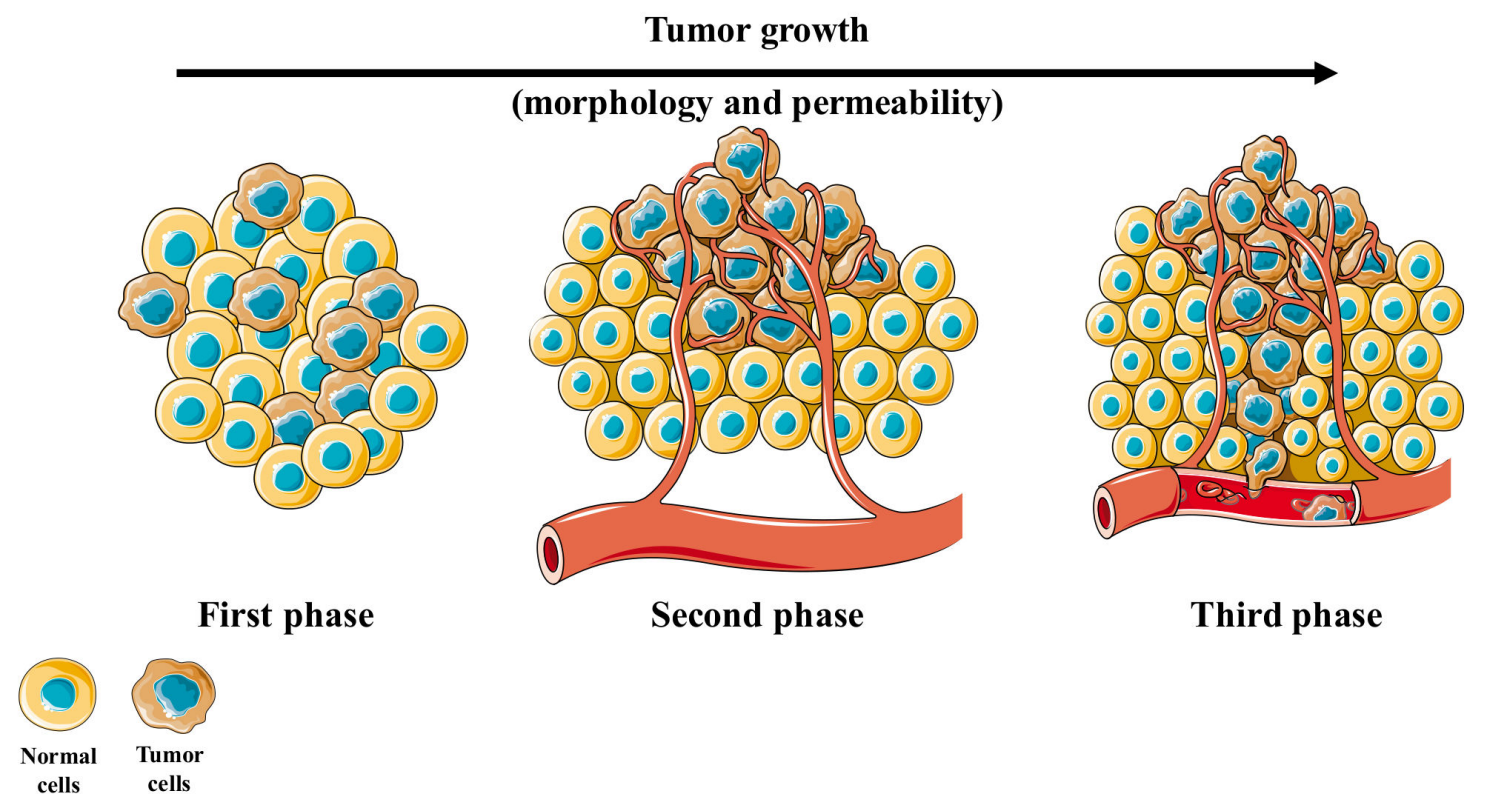
Morphology and permeability phases of the BBTB.

The status, function and organization of the BBB is different depending on the brain disease. Barrier modification, in morphology and permeability, occurs depending on the progress of tumor. Thus, GB, a high-grade brain tumor, induces major alterations characterized by abnormal vascularization, resulting in a disrupted blood–brain–tumor barrier (BBTB). The BBTB is designed by brain tumor capillaries and forms a barrier that is different from the BBB. In addition, tight junctions of endothelial cells in a brain tumor scenario are compromised (Figure 3). The high metabolic stresses of GB produce hypoxic areas that have an increased expression of vascular endothelial growth factor (VEGF) and angiogenesis, with a dominant formation of abnormal vessels and, consequently, a dysfunctional BBTB. The fenestration in BBTB displays a significantly higher permeability compared to the BBB, allowing molecules with size below 12 nm to pass from the blood stream into the brain. Depending on the GB phase, there are regions in the brain that present fenestrations whose size is variable and can increase to a micron, more specifically in the third phase (Table 4). Thus, the BBTB is susceptible for nanocarriers and enhanced permeability and retention (EPR) effect, with a preferential accumulation in the tumor tissues [132,133]. However, the rapid progression of GB forced by the tumor aggressiveness, leads to a quick spread into the surrounding healthy tissues causing the BBTB damage and prompting EPR effect. The BBTB remains a hurdle for drug delivery, leading to heterogeneous drug accumulation, which compromises the therapeutic outcome. BBTB shows specific characteristics and overexpressed receptors that mediate ligand dependent drug delivery, which can be exploited to selectively enhance drug delivery to tumor tissues. Anti-angiogenic therapies are being applied as an adjuvant to regulate GB vasculature.

BBTB blood vessels contribute to the delivery of nutrients and oxygen to the tumor and accelerate glioma cell migration to other parts of the brain. Some overexpressed receptors on BBTB are receptor tyrosine kinases (RTKs). RTKs are described as high affinity cell surface receptors and mediate signal transductions with an essential function in the growth and progression of GB. VEGF receptors, epidermal growth factor receptors (EGFR) and platelet-derived growth factor receptor (PDGFR), provide a chance for glioma targeting drug delivery.

EGFR is a receptor tyrosine kinase and the most frequent amplified gene in GB, being responsible for the radiotherapy resistance. Activation of these receptors results in activation of multiple signaling pathways (e.g., the Ras/Raf/MEK/ERK1/2-mitogen-activated protein kinase (MAPK) pathway and the phosphatidylinositol 3′-kinase(PI3K)/Akt/mTOR pathway) [136]. EGFR plays a critical role in the development and progression of the diseases, affecting cell proliferation, migration, differentiation, inhibition of apoptosis and increased angiogenesis. 

VEGF receptors (VEGFRs), including VEGFR-1, VEGFR-2, and VEGFR-3, are overexpressed in ECs to control tumor vasculature. As mentioned in Section 2.1.4., the high metabolic stress creates hypoxic areas, which lead to an increased expression of VEGF and angiogenesis. VEGF is one of the most crucial growth factors and plays an essential role in GB neovascularization by interacting with a number of signaling pathways, so targeting VEGF pathways would reduce tumor vasculature [134,135]. The identification of the VEGF pathway is a key regulator of angiogenesis. 

#### 2.1.3. Tumor Microenvironment

Tumor growth and progression are caused not only by cumulative gene mutations, but also by the significant influence of the surrounding tumor microenvironment (TME) [137]. TME is a dynamic space and is characterized by cellular heterogeneity, including endothelial cells (ECs), extracellular matrix (ECM), macrophages, fibroblasts, perivascular cells, and inflammatory cells [138]. All of these components are the key for governing cellular growth, the preservation of homeostasis, being involved in the regulation of the tumorigenic process (angiogenesis, lymphanogenesis, and inflammation). The TME has significantly contributed to the failure of conventional chemotherapy, and to the malignant progression, dictating the incomplete eradication of the tumor [139]. Tumor progress requires constant maintenance, which is dependent of the interaction of the immune cells and the TME. Consequently, cancer cells can control the immune cells so as to promote cancer development and related pathological processes. TME is likely to influence malignant cell growth by releasing proteins, growth factors, cytokines, and proteases that allow cancer cells motility and adhesion. In fact, the understanding of the function of the TME in tumor progression is critical to cancer eradication. Strategies that inhibit this support to tumor cells reduce chemoresistance and tumor recurrence [137,140,141]. These consider the modulation of the tumor extracellular matrix (ECM), immune cells and response to tumor hypoxia. Although there has been considerable progress in the use of nanoparticles directed at the TME, these new strategies still face many challenges in terms of their clinical impact.

The extracellular matrix (ECM) comprises all secreted soluble and insoluble components found in the extracellular space, e.g., collagen, elastin fibers, glycoproteins, proteoglycans, and hyaluronic acid (HA) [142]. ECM components and their complementary receptors (e.g., integrins and CD44) not only contribute to cellular organization, but also to cell behavior and brain tumor evolution, with an important role in processes, such as proliferation, survival and differentiation of cells. The cell development is ensured by ECM functionality, which provides the maintenance of structural integrity and the transport of nutrients and oxygen. Thus, ECM is multifunctional and can stimulate several mechanical and biochemical processes simultaneously, including an adhesive substrate which provides structure, present growth factors to their receptors, senses and transduces mechanical signals sequesters and stores growth factors. The highly-upregulated ECM molecules in TME have been extensively correlated with the malignancy of the tumor. Lactic acid, fatty acids, angiogenic growth factors, stromal cell-derived factor 1, angiopoietin 2, tenascin-C or connective tissue growth factors are some examples of ECM molecules which accumulate in the tumor microenvironment [135,143].

Protease and lipase are enzymes overexpressed by tumor cells that can be used as endogenous triggers for cancer targeting. Matrix metalloproteinases (MMPs), especially MMP-2 and MMP-9, are responsible for the changes in TME during tumor development. They mediate ECM degradation and support cancer cell metastasis and invasion. MMPs also regulate signaling pathways that control cell growth, inflammation, or angiogenesis. 

The interaction of glioma cells with the ECM is largely mediated by mediated by cell surface receptors of the integrin. Integrins are extracellular matrix transmembrane receptors, including α6β4, α5β1, αvβ6, and αvβ3, that are overexpressed on GB, but not on normal brain tissues, and this represents a typical targeted treatment strategy directed to angiogenesis, tumor cell proliferation, inflammation and survival [144]. αvβ3- and αvβ5-associated angiogenesis are respectively dependent on tumor cell-secreted fibroblast growth factor (bFGF)/tumor necrosis factor (TNF)α and VEGF through an amplification loop leading to αvβ3/αvβ5 overexpression on EC [145,146,147,148,149,150]. Integrin-mediated signaling pathways have been found to promote the invasiveness and survival of glioma cells in brain microenvironment, more concretely they support the formation of the tumoral niche [151]. As such, integrins are a promise targeting for GB treatment due to their implication in tumor cell functions and their interaction with several pathways, including MAP kinase, Jun, NF-κB, and their location at the TME [110,145,152,153].

CD44 is a transmembrane glycoprotein expressed on normal cells and overexpressed in glioma cells [154]. CD44 is a cell membrane receptor that mediates cell–cell and cell–ECM interactions. CD44 serves as a surface receptor for ECM molecules such as hyaluronic acid (HA) and chondroitin sulfate proteoglycan (CSPGs). The HA–CD44 interaction and CD44 shedding from the cell surface were found to be associated with glioma cell motility, migration, and infiltration into the normal brain parenchyma [155]. 

Different types of immune cells, including T and B lymphocytes, natural killer T (NKT) cells, dendritic cells (DCs), tumor-associated macrophages (TAMs), tumor-associated fibroblasts (TAFs) and myeloid-derived suppressor cells (MDSCs) can infiltrate in the TME. GB is associated with several immunosuppressive cytokines. These molecules are secreted by immune cells and are responsible by blocking T-cell activation and proliferation, inhibiting IL-2 production, suppressing natural killer cell activity, and stimulating regulator T cells (Tregs), and support tumor growth, invasion and enhance angiogenesis [156]. Thus, targeting tumor-associated immune cells is described as an interesting strategy, due to the stimulation of immune response against cancer cells. Vaccination, checkpoint inhibitor molecules, adoptive cell therapy, and monoclonal antibodies are some examples of immunotherapy strategies. Some of them have already been recognized as an advantageous treatment [157].

**Figure 4:**
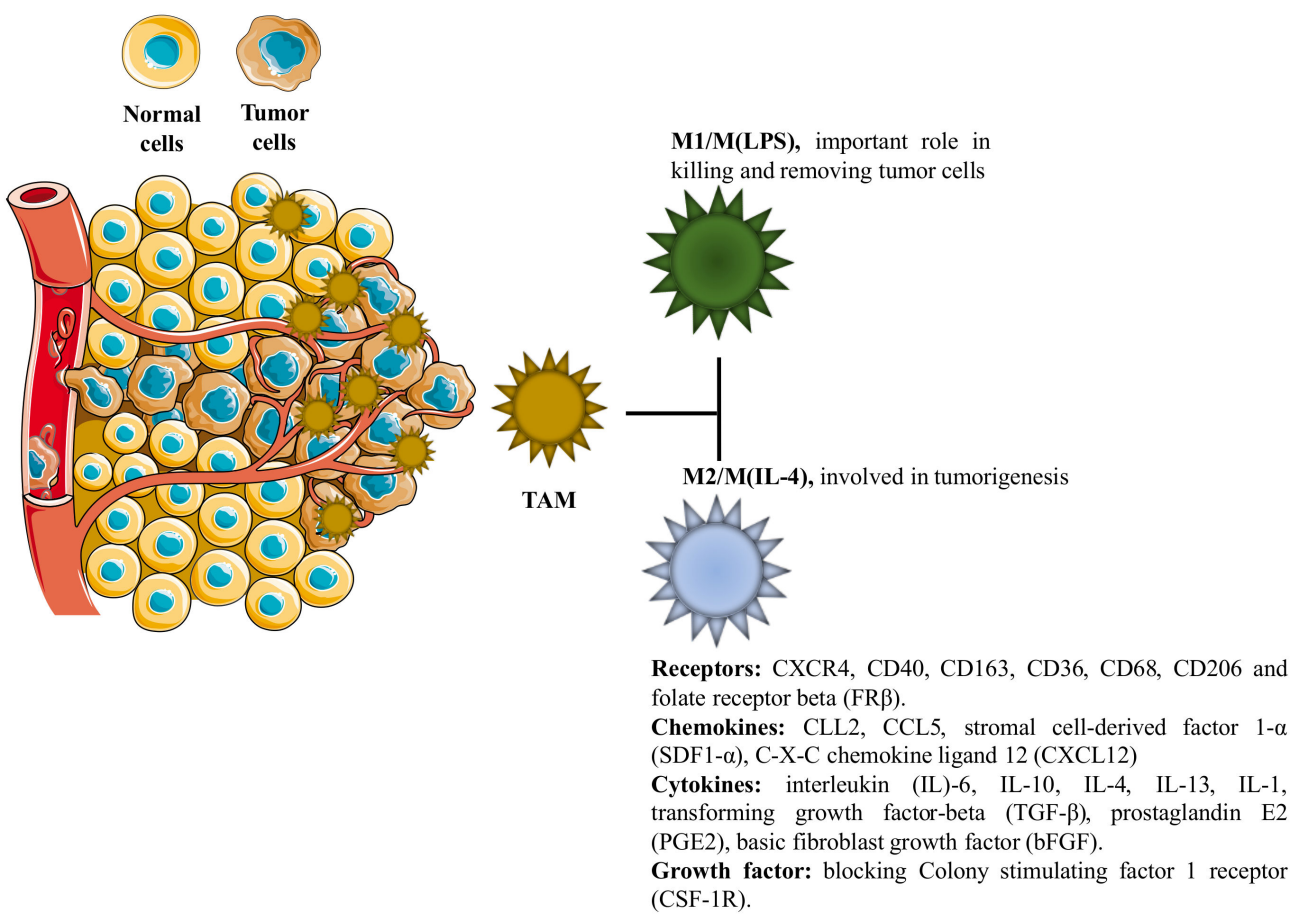
Tumor-associated macrophages (TAMs) in tumor microenvironment: macrophages phenotypic differentiation into either M1 or M2 subtype. M2 is the subtype of TAM involved in tumorigenesis and description of receptors, chemokines, cytokines and growth factor as targeting approaches [161].

Tumor-associated macrophages (TAMs) are inflammatory cells of the innate immune system, which represent ca. 30–40% of the cells in GBM; their presence has been correlated with poor prognosis in cancer [158]. Recent work has also addressed the effect of mesenchymal stem/stromal cells (MSCs) that constitute an attractive tool for cell-based cancer therapy mainly because of their ability to migrate to tumors and to release bioactive molecules [159,160]. TAMs in tumor microenvironment promote immune evasion and angiogenesis, stimulate cancer cell proliferation, immunosuppression, and support tumor growth and metastasis. TAMs are also described as a possible cause for drug resistance with radio protective effects, having been associated to therapy failure [161]. There are essentially two types of macrophages: one supporting the inflammatory response and antitumor immunity (M1/M(LPS)), having an important role in killing and removing tumor cells, and the other involved in tumorigenesis (M2/M(IL-4)), being associated to pro-tumorigenic activities angiogenesis, recruitment of leukocytes, and immune suppression [162,163]. TAMs targeting is a promising adjuvant approach for anti-cancer treatments (Figure 4) [162,164,165,166,167]. Indeed, different research groups have found that the NPs accumulate at high levels within TAMs, and that TAMs serve as “cellular drug reservoirs” [166,168,169,170]. Consequently, there is a reduction of actively targeted NPs to the target sites, and a significant decrease of the intratumoral NPs density. Due to the importance of TAMs, they are becoming the main target of new therapeutic strategies, and can be adopted for a future development and clinical use of NPs. As previously stated, TAMs are an important component in TME and execute tumor-protecting functions. Thus, a higher NPs accumulation in TAMs can be considered as an appealing strategy for cancer treatment, especially relevant in the context of therapies that increase or decrease infiltration of macrophages to tumors [171]. GB-associated macrophages are mainly bone marrow (BM)-derived infiltrating myeloid cells [172,173]. These cells arise in an early stage of tumor formation, and their location in the perivascular niche contribute to tumor progress. Recent studies showed that reducing their infiltration by genetic modulation significantly prolongs the survival in mice [173].

Fibroblasts and bone marrow-derived mesenchymal stem cells are activated through growth factors released by tumor cells in tumorigenic process [174]. Tumor-associated fibroblasts (TAFs) promote tumor development by secreting cytokines as VEGF, IL-6, and HGF, that are responsible by the induction of the tumor vascularization and subsequently stimulate cell proliferation. TAFs produce a high amount of proteoglycans, collagens and secrete different extracellular enzymes, such as MMPs, disintegrins, and plasmin [175].

The decrease of nutrient and oxygen transport by abnormal tumor vasculature results in hypoxic sections, which are commonly evidenced within most solid tumors. Hypoxia has correlation with the insufficient blood flow, while chronic hypoxia is the outcome of amplified oxygen diffusion distance [176]. The critical pathogenetic mechanisms for the development of hypoxia are structural and functional abnormalities in the tumor microvasculature, an increase in diffusion distances and tumor- or therapy-associated anemia leading to a reduced O_2_ transport capacity of the blood, which can stimulate an adaptive reaction [177]. The extent hypoxia depends on clinical size, stage, and grade. Therefore, this adaptive mechanism leads to a negative impact in radiotherapy and chemotherapeutics by reducing the oxygen level needed for free radical formation, which is critical for cell cancer death [178]. The hypoxia leads to the activation of the phosphatidylinositol-3 kinases (PI3K)-Protein Kinase B (Akt) pathway. Akt is involved in several cellular functions including cell proliferation, apoptosis resistance, cell survival and a diversity of metabolic functions. Thus, the activation of those pathways allows the activation of the hypoxia-inducible transcription factor 1 (HIF-1), a heterodimeric protein [179]. In normal tissues, HIF-1a is destroyed by the ubiquitin-proteasome system, but when the intracellular oxygen concentration decreases, HIF-1a is stabilized and translocated to the nucleus where it binds to HIF-1b. The HIF-1a and -1b dimers activate transcription of genes involved in angiogenesis, glucose transport, apoptosis resistance, metastasis and inflammation [180]. The pro-inflammatory components include membrane receptors RAGE and P2X7R; inducible enzymes, e.g., COX-2 and NOS-2; and proteins such as PTX3 [180]. Moreover, the hypoxia environment allows an increase of expression of chemokine (C-X-C motif) receptor 4 (CXCR4), one of the components responsible for the invasion and migration of tumor cells. Several approaches for targeting hypoxic tumor cells have been suggested, including gene therapy, specific targeting of HIFs, or targeting important pathways in hypoxic cells [181,182]. A consequence of hypoxia is the conversion of glucose into lactate to produce ATP, CO2 and carbonic acid. The low clearance of these acidic metabolic products causes a decrease in interstitial pH of solid tumors, including GB [183]. For this reason, the extracellular tumor pH (pHe) is around 5.7–7.2. To take advantage of this characteristic, different approaches have been developed, including pH-triggered-NPs, which increases drug accumulation inside the tumor and reduces the cytotoxicity to the normal tissues [184].

#### 2.1.4. Glioma Stem Cells

**Figure 5:**
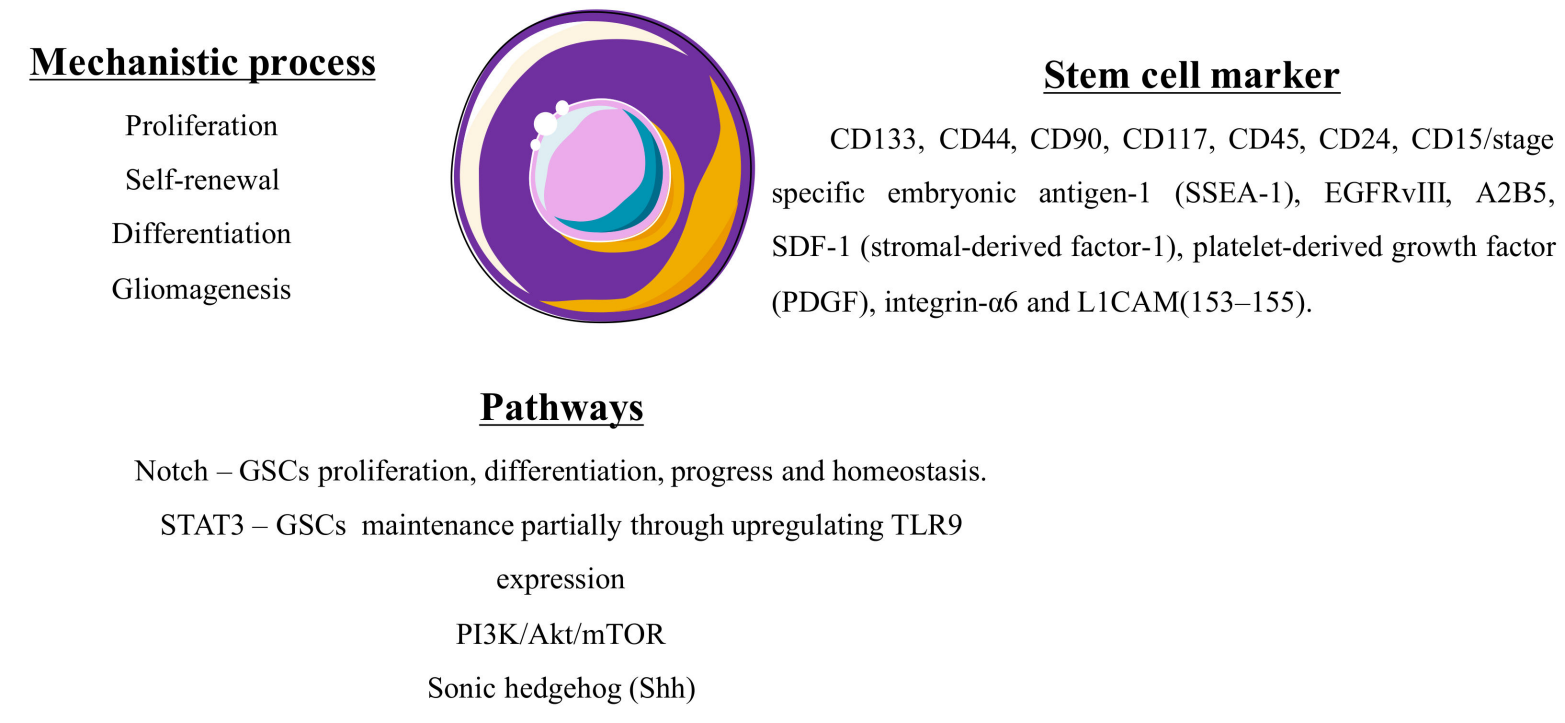
Glioma stem cells (GSCs) mechanistic process, including proliferation, self-renewal, multidifferentiation and tumorigenic capability, and possible surface markers/pathways for targeting approaches [145,185,186,187,188,189,190].

Glioma stem cells (GSCs) characterize a subpopulation of tumor cells within GB which are possibly the cause of the increased resistance to conventional therapies, and, consequently, the major cells responsible for failure of treatment and high recurrence rates in GB. GSCs present the same characteristics of normal stem cells with the particularity of being oncogenic in their host and giving rise to a heterogeneous population of cells that comprise the tumor mass. Proliferation, self-renewal, multi-differentiation potential in vitro and tumorigenic capability in vivo are some of their mechanistic process (Figure 5). 

**Figure 6:**
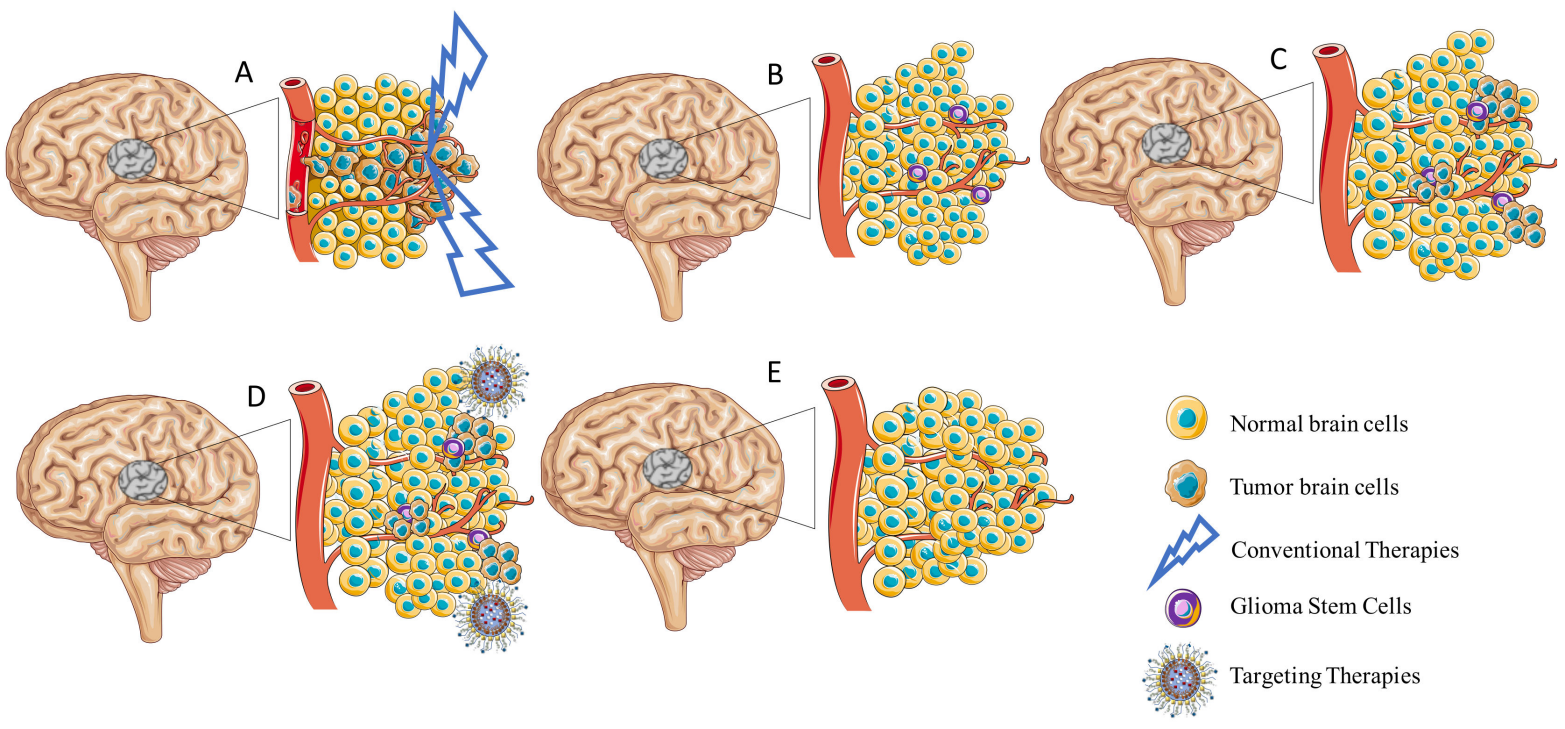
Influence of the targeting approach on GSC function. The application of conventional therapies (**A**) lead to resistance of GSCs (**B**). GSCs cells, which are present in the specific perivascular niches, restart their growth process and develop tumor cells, due to their high number of surface markers, differentiation markers, and distinctive stimuli, such as cytokines, growth factors and the angiogenesis process (**C**). In this context, a targeting approach strategy involving the binding to the surface markers or cytokines, which are responsible for tumor growth or cytokines inhibition, may be an excellent strategy against GSCs (**D**), avoiding tumor recurrence (**E**).

Considering all these mechanisms, GSCs contribute to tumor beginning and therapeutic resistance, so this is one of the main reasons of the failure to eliminate the tumor cells (Figure 6) [191]. To improve the conventional therapies, it is possible to take advantage of stem cell marker expression. Thus, targeting cell surface markers and specific pathways that are essential for maintaining GSC represents a smart and important strategy for therapy of GB (Figure 5) [192,193,194]. The niches where GSCs reside are organized by immune cells, fibroblastic cells, endothelial cells, integrin receptors, ECM components, cytokines and growth factors. As such, targeting GSCs and their niches introduce the possibility to eradicate the likely basis of gliomas and their recurrence chance [158].

## 3. Strategies to Enhance the Permeability of the Blood–Brain Barrier for Treatment of Glioblastoma

**Figure 7:**
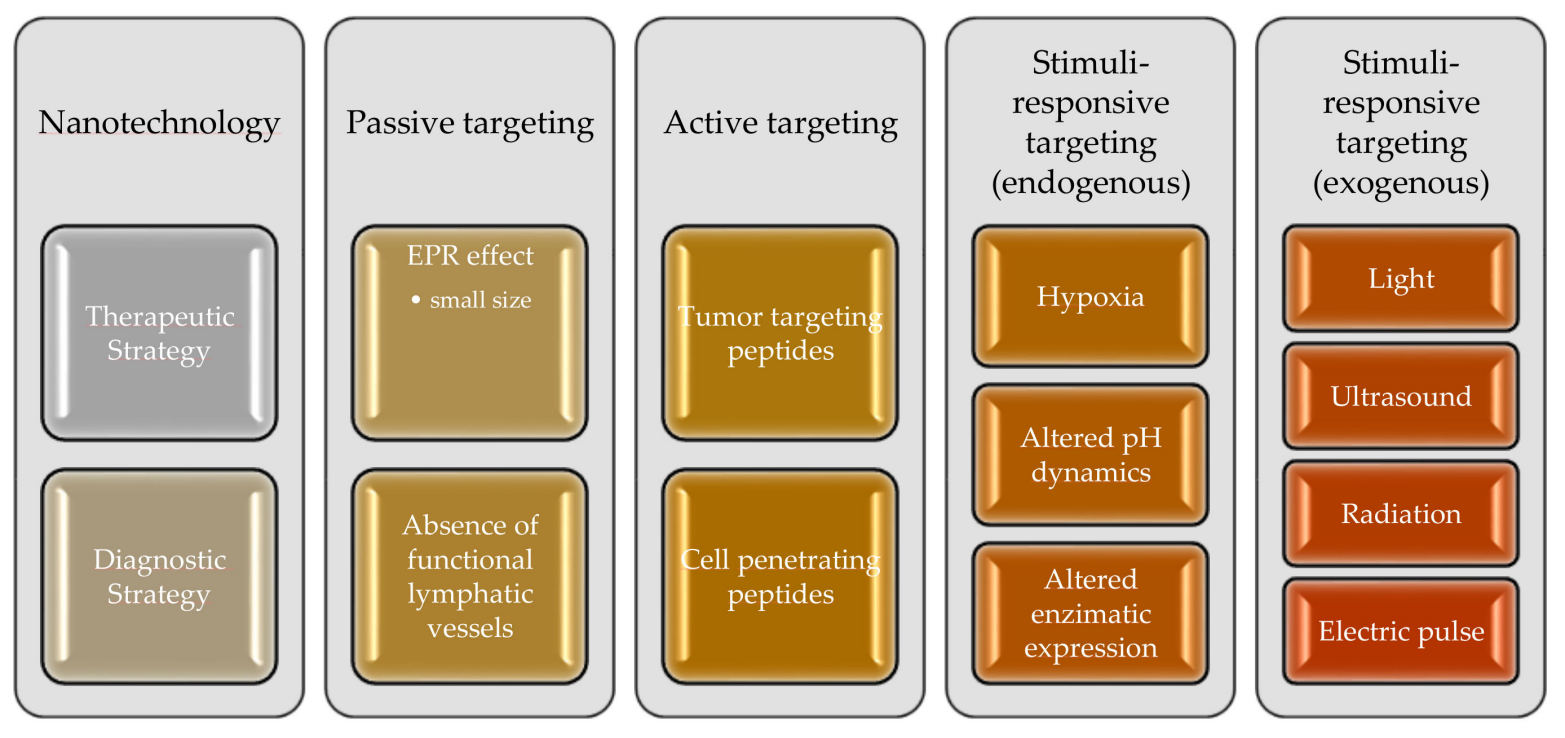
Strategies to enhance the permeability of the blood–brain barrier for treatment of GB.

The heterogeneity of GB, in combination with its high infiltrative nature, determine a poor outcome for standard treatments. To overcome these hurdles, different targeting strategies have been adopted for developing effective nanostructured delivery systems (NDS) to the brain. There are many strategies described to direct NPs to the brain tumor, including the use of nanotechnology coupled with passive, active or stimuli responsive targeting approaches (Figure 7) [195,196,197,198,199].

### 3.1. Nanoparticles for GB Treatment

**Figure 8:**
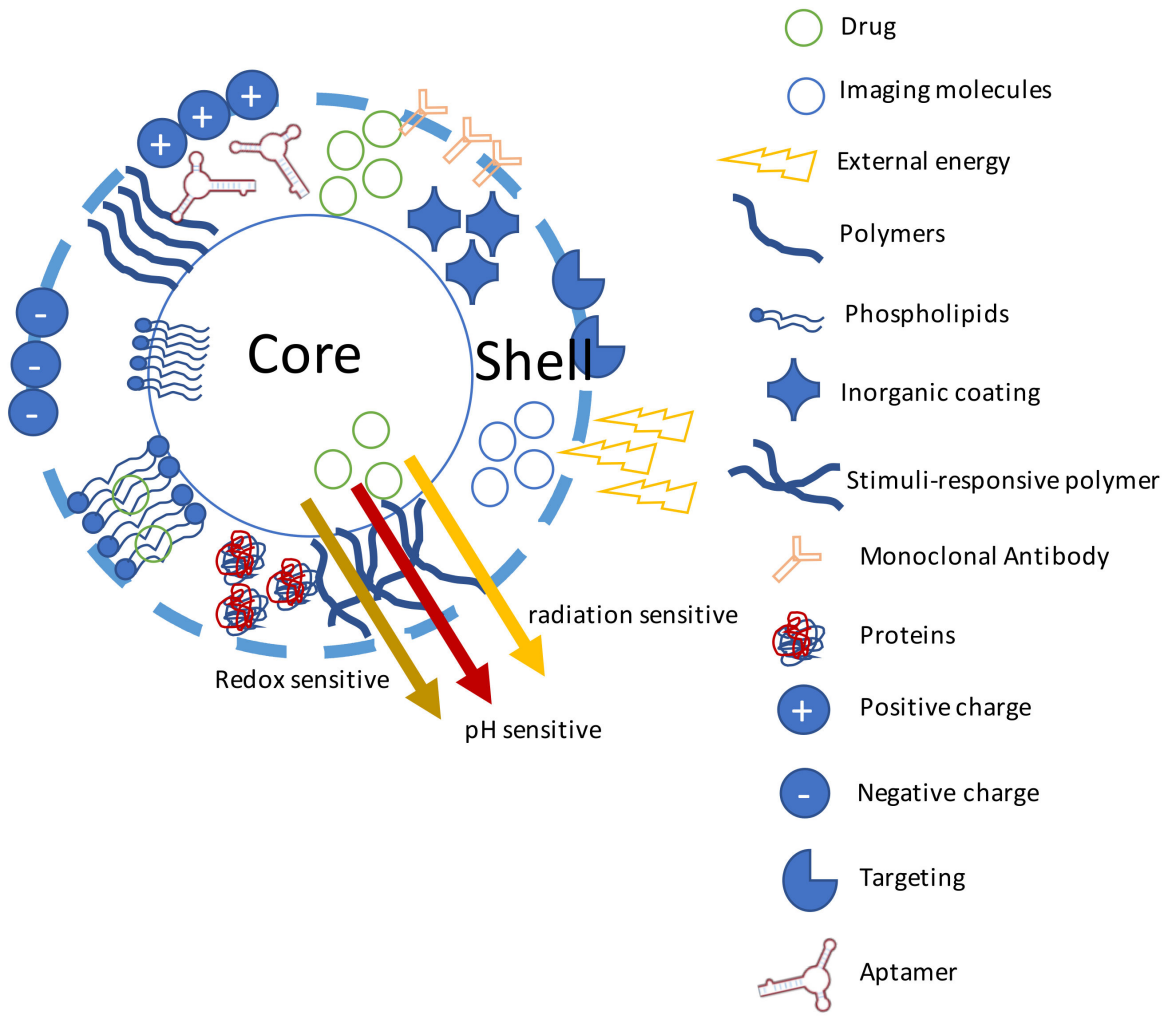
Schematic representation of core–shell nanostructures and possible modifications (Reprinted from [260] with permission from Elsevier, 2018).

Nanotechnology provides endless opportunities in the area of cancer treatment using targeted anticancer drug delivery systems. For that, NPs have to be appropriately studied and designed to accomplish the greatest therapeutic and/or diagnostic effect with reduced side effects, especially for chemotherapy drugs [200] (Figure 8). NPs are complex drug delivery systems, which can be structurally divided into the external layer (shell) and internal layer (core). The shell can be functionalized with a variety of small molecules, polymers, polysaccharides, proteins and metal ions; the core, a central part of the NPs, can be chemically combined with different materials. NPs are flexible and versatile, with an architecture that ensure small size, appropriate shape, and surface functionalization, in order to accomplish the objectives of the proposed function. NPs have been shown to improve pharmacokinetics of available drugs, by reducing drug biodistribution to non-target compartments, delivering the adequate amount of the drug into the right site, overcoming solubility and stability issues and allowing higher drug encapsulation. Regarding the complexity of BBB and/or BBTB, NPs offer the possibility to encapsulate hydrophilic anticancer drugs, increase the blood circulation and tissue distribution, with improvement of their preferential accumulation at the tumor site. Additionally, NPs have been described to overcome multi-drug resistance mechanisms [201]. Several nanostructured delivery systems (NDS) have been developed for brain tumor delivery, and depending on the nature and composition, they can be classified as organic NPs and inorganic NPs [202,203]. Organic NPs are described as being composed by organic compounds, including lipids, surfactants or polymers with the GRAS (generally regarded as safe) status, that offer an easy way for the encapsulation of molecules. Liposomes, polymeric nanoparticles, lipid nanoparticles, micelles/polymeric micelles, protein based nanoparticles are some examples of organic NPs. An extensive research is already reported with organic NPs for the GB treatment. The NPs performance has been assessed through in vitro and in vivo studies, and compared with the free drugs, and the results seem to be promising, showing the ability to transport drugs across the BBB in a more efficient way, with a preferential distribution in the brain [76,146,149,204,205,206,207,208,209,210,211,212,213,214,215]. On the other hand, inorganic NPs contain a solid core with physicochemical properties that can be attributed to their inorganic components, such as magnetic metal oxide or semiconductor material. Different types of inorganic NPs have been described and employed for biological applications, including iron oxide nanoparticles, mesoporous silica nanoparticles, gold nanoparticles and quantum dots. The main advantages of inorganic NPs are their robustness, resistance to enzymatic degradation and interesting intrinsic characteristics, such as optical, thermal, magnetic and electrical properties, that can be used for imaging and therapeutic approach. Considering their nature composition, inorganic NPs can be synthesized and modified in a way that facilitates the surface functionalization by incorporation of ligands or polymers, improving their biological function [216]. The use of biocompatible coatings has shown to reduce the toxicity associated to the presence of the heavy metal. Inorganic NPs have also been widely investigated for diagnostic applications, using their distinct abilities to respond to external stimuli and physiological changes [217,218,219,220,221,222,223,224,225,226,227,228,229,230,231,232,233,234,235,236,237,238,239,240,241,242]. As shown in different reviews, several types of NPs (magnetic, fluorescent, liposomal, polymeric, lipidic, among others) have already been designed and developed for crossing the BBB to GB therapy, considering passive, active and stimuli targeting approaches [6,133,243,244,245,246,247]. NPs are an interesting drug delivery system, presenting a high potential for improving patient outcomes. However, their translation from pre-clinical proof of concept to the demonstration of therapeutic value in the clinic remains a long, costly, and challenging path. The scaling up procedure, surface modification, biopersistence and toxicological aspects, recognition and quantification of NPs in the human body are some critical points that make difficult the translation. Several types of research focusing on NPs characterization, and in vitro and in vivo studies have been carried out, but many questions remain without answers. In vitro and in vitro studies for GB treatment, encompassing multiple biological targets, have been described and include gold nanoparticles, chitosan-based nanoparticle, curcumin-loaded PLGA-DSPE-PEG nanoparticles, curcumin-loaded RDP-liposomes, hyaluronic acid conjugated liposomes, iron oxide nanoparticle coated with chitosan-PEG-polyethyleneimine copolymer and CTX loading plasmid DNA encoding TRAIL, c(RGDyK)-pHA-PEG-DSPE-incorporated DOX loaded liposomes, and others [248,249,250,251,252,253,254,255]. These have been considered as effective against glioblastoma, and in vivo results show a potential application in diagnosis, with important therapeutic implications, resulting from a significant accumulation in the brain tumor regions. Only a small number of NPs have been evaluated in clinical trials, and fewer were extended to clinical practice [256]. In what follows, the reference of clinical trial is quoted, as extracted from clinicaltrial.gov. REF Nanoliposomal CPT-11 (NCT00734682 and NCT02022644), SGT-53 (liposomal nanocomplex encapsulating a wild type p53 DNA sequence) used in combination with temozolomide, NU-0129 (NCT03020017, gold nanoparticles based on spherical nucleic acid) and PEGylated liposomal DOX (NCT02766699, Caelyx^TM^, PEG-Dox) constitute some of the clinical trials that have been conducted in recent years, and illustrate the variety of theranostic approaches. NanoTherm (Aminosilane-coated SIONPs) and Opaxio (ester conjugate of α–poly(l)–glutamic acid (PGA) and paclitaxel) are NPs already implemented in clinic. However, the inability to effectively control the behavior of NPs inside the body, including biodistribution, toxicity and pharmacokinetics, represents a major limitation for using nanotechnology to diagnose and treat cancer [257,258,259].

### 3.2. Passive Targeting

Passive targeting resorts to brain tumor properties, including hyper-vascularizing, leaky and scarce lymphatic drainage system, to make drug available into the intratumoral space, while depriving healthy brain tissue. NPs dynamics through the brain depend on the integrity of the barrier, which may be compromised in the presence of some diseases, and on the size, geometry and surface properties of the actual particles. These parameters must be controlled to avoid uptake by the reticuloendothelial system (RES). To maximize circulation times and targeting ability, literature indicates NPs optimal size as less than 100 nm in diameter, and suggests the presence of an hydrophilic surface to circumvent clearance by macrophages within the RES [261]. Nanoparticles 100–400 nm in size did not show significant difference for overcoming the BBB. However, significant differences were observed when nanoparticle size was below 100 nm, for which the effects were more pronounced [262]. Some work focused on correlating the size of NPs to their biological effects [263,264,265]. It can be concluded that the size and the charge of nanoparticles are controversial in the effects promoted in vivo, and further studies are needed to clarify this subject. Thus, NPs have the advantage of the small size to provide a preferential accumulation at the site of brain tumor due to the morphology and permeability of the barrier, EPR effect (Figure 6) [201,266]. The EPR effect does not occur with the conventional chemotherapeutic molecules, due to the lack of specificity between normal and tumor tissues. However, the ability of drug diffusion is an important parameter that hinders the passive targeting strategies, due to the difficulty to control drug release. Therefore, to achieve an efficient EPR effect, NPs should be smaller than 100 nm and present a biocompatible surface (neutral charge and hydrophilic properties) to avoid the reticuloendothelial system (RES) [40,267].

### 3.3. Active Targeting

To increase the selectivity of drug release at the chosen sites of action, and achieve enhanced therapeutic efficacy, an active targeting approach is required [268]. These strategies consist in incorporating affinity molecules or taking advantage of influx transport systems expressed within the BBB/BBTB, into NPs surface, bearing in mind the specific characteristics of abnormal tissue, e.g., the differential expression of receptors or antigens in cancer cells (as described in Section 2.1). These include a wide range of peptides, antibodies or antibody fragments, aptamers and other small molecules [62,72,254,269,270,271]. GB is characterized by several molecular mechanisms of chemoresistance, so the development of actively targeted NPs to surface cell markers, signaling pathways and tumor microenvironment represents an interesting and challenging opportunity (Table 5). These approaches are accomplished by connecting specific ligands to the NPs structure, which allows a selective recognition of different receptors overexpressed in the tumor cell surfaces. The functionalization of the NPs surface improves therapeutic efficacy of cytotoxic drugs and overcomes the multidrug resistance (MDR).

### 3.4. Stimuli-Responsive Strategies

The lack of control over drug release has prompted the development of NPs tailored to respond to endogenous and/or exogenous stimuli (Figure 8). The benefits of internal-stimuli responsive NPs are evident, because the stimuli specifically exists in characteristic pathological sites [272,273,274]. The endogenous triggers, such as pH variations, enzymes, glucose or redox gradient, can be used depending on the disease pathological characteristics [275]. As previously discussed (Section 2.1.4.), tumors have an acidic pH in contrast to the normal tissues, so there is an opportunity to use pH-stimuli responsive NPs [276,277,278]. Due to their conformation changing or bond cleavage sensitive to different pH values, pH-responsive NPs allow the release of loaded drug in precise locations. This strategy has been applied by different research groups, and the results have been satisfactory [96,102,113,127,128,129,130,131,279]. The redox-sensitive NPs are used also as a stimuli response, due to the intrinsic redox gradients in the cells. Taking advantage of the higher concentration of glutathione (GSH) that regulates the intracellular redox condition, NPs with disulfide bonds are broken down and the cargo is released. This bond is unstable in high concentrations of GSH, being reduced to thiol groups inside the cells. The enzymatic-stimuli response is also an interesting approach, due to the high expression of enzymes in the tumor bulk, in particular extracellular matrix metalloproteinases (MMPs) [124,125,126,280,281,282].

In turn, the exogenous triggers use external forces, including temperature, magnetic field, ultrasound, light, electric pulse, or high radiation [283]. Targeting moieties can be combined directly or indirectly by covalent or non-covalent linkage.

The heterogeneity in the physiological conditions, the combination of endo- and exogenous stimuli-responsive NDS might be more favorable, allowing to target different environments. It is possible to combine triggers, such as pH/temperature, pH/redox, temperature/redox, and temperature/pH/redox, among others, which exhibit additional advantages in comparison to NDS employing single stimulus. This is a field that has attracted much attention in recent years [275,284].

## 4. Theranostics

**Scheme 1:**
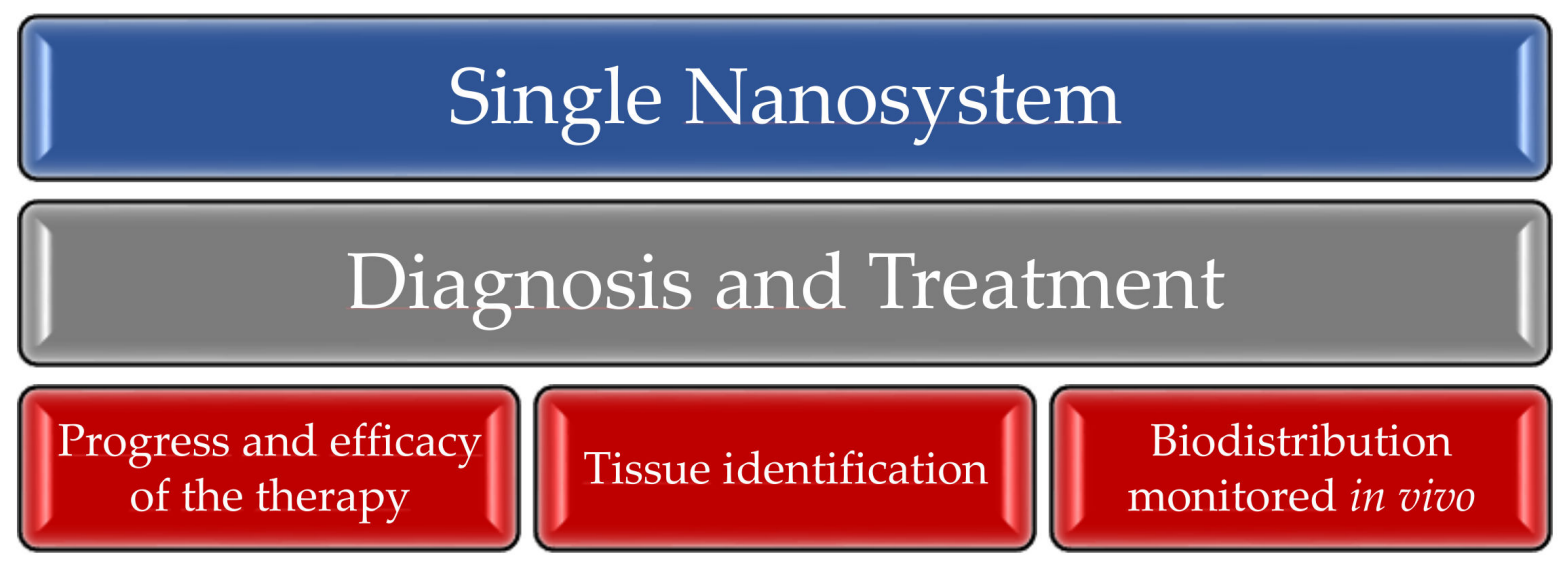
The main goal of the use of theranostic NPs.

The current progress in GB therapy and detection evidences that there has been no significant reduction in GB associated with death rates. The heterogeneity of the tumor, the unspecific drug delivery, the diagnosis failure in the primary stage detection are some of the reasons responsible for the treatment inefficiency. Thus, the combination of earlier detection and targeting therapy procedures, termed as theranostics, has been developed as a strategy to improve the GB treatment. The theranostic approach make possible to diagnose the GB, understand the location of the GB and the stage of disease, and also to percept the tumor progression. These strategies help to address the intra- and interpatient heterogeneity of GB, pointing out to a future application in personalized medicine. The use of a single and multimodal system appears to be one of the most promising characteristics of NPs application (Scheme 1).

**Figure 9:**
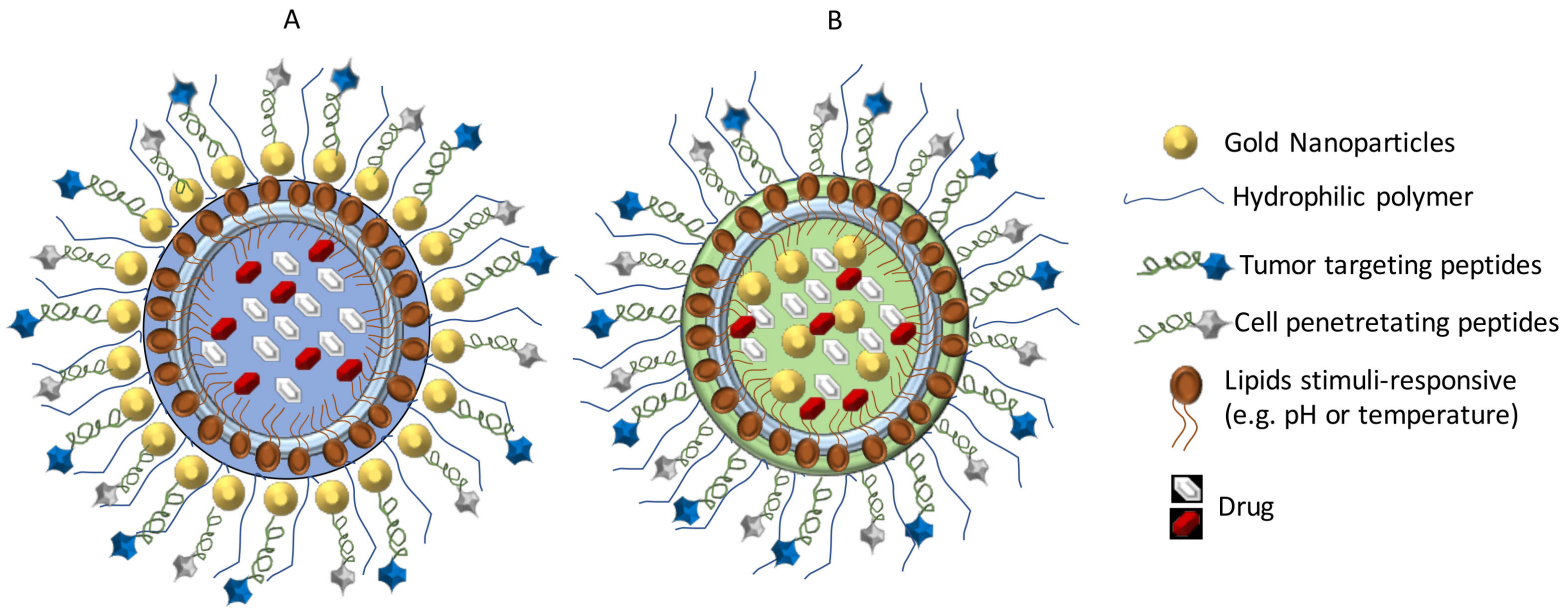
Two prototypes of theranostic nanoparticles, combining lipid nanoparticles and gold nanoparticles, with characteristics such as small size (<100 nm), active targeting (tumor targeting and cell penetrating peptides) and a stimuli-responsive targeting (pH or temperature): (**A**) AuNPs attached on the NPs surface by electrostatic interaction; and (**B**) AuNPs with lipophilic characteristics encapsulated in NPs.

In this context, a new technological concept has been introduced and designed as theranostic nanoparticles (TNPs) (Figure 9). TNPs are described as a combination of both organic and inorganic NPs to obtain multiple synergistic properties in a single NP, taking advantages of the drug delivery by organic NPs and imaging prompted by inorganic NPs [374,375,376,377]. The preparation methods and physicochemical characterization through in vitro evaluation on cell culture and in vivo studies have been described for a variety of TNPs. These NPs have shown interesting results during in vitro studies. However, considering the different diagnostic approaches, including magnetic resonance imaging (MRI), computed tomography (CT), ultrasound, optical imaging (OI) and photoacoustic imaging (PAI), positron emission tomography (PET) and single photon emission CT (SPECT), there are still some challenges for their application in vivo. The imaging modalities are based on diverse physical principles, considering the method that allows the higher sensitivity and specificity to tissue contrast, quantitative and tissue penetration, and spatial resolution. Consequently, the TNPs concept needs an extensive in vivo investigation before their clinical translation.

In fact, several inorganic and organic NPs utilized in the diagnosis and drug delivery have been developed for different applications. Theranostic nanoparticles have shown attractive results in in vitro studies, but there are still some challenges for their application in vivo. For this reason, the major part of the research that has been described in the literature is about the preparation and characterization of TNPs with application in in vitro cell culture, still lacking the in vivo proof of concept. In the case of the treatment of human brain cancers, TNP have been developed [378,379,380,381,382,383], taking advantage of the specific GB characteristics described in the following sections. Gold and iron oxide-based nanoparticles have prompted particular attention as theranostic anti-cancer agent systems, combining in the same NPs drug delivery, imaging, and therapy [384,385]. Thus, TNPs can be used to avoid frequent and invasive dosing and improve patient compliance. Recently, a research group combined chemo-photothermal targeted therapy of glioma within one nanoparticle. A targeting peptide (IP)-modified mesoporous silica-coated graphene nanosheet (GSPI) was synthesized and characterized. Doxorubicin (DOX) was used as therapeutic component and GSPI nanoparticles as drug and diagnostic delivery system, integrating the response to heat and pH-stimuli. Only in vitro studies were performed and the results showed a higher rate of death of glioma cells and improved accumulation of GSPID [228]. In another work [386], targeted TNPs were developed, which included newer (small interference RNA, siRNA) and conventional (temozolomide, TMZ) therapeutics. To promote the targeting to glioma cells, chlorotoxin (CTX) peptide was conjugated with iron oxide nanoparticles that worked as drug carrier and allowed to monitor the changes in tumor volume by magnetic resonance imaging (MRI). TNPs were internalized by T98G glioblastoma cells in vitro leading to the enhancement of TMZ toxicity. The results in vivo indicated that the combination of the treatments with the TNPs loaded with TMZ led to significant retardation of tumor growth, as monitored by MRI. The multiple emulsion solvent evaporation method has been proposed as effective for high co-encapsulation of SPIONs and DOX into the poly (lactic-co-glycolic acid) (PLGA)-based NPs. A different strategy was designed, taking advantage of magnetic nanoparticles and polymeric materials for potential application in targeted therapy and imaging of malignant tumors. Thus, SPIONs and DOX were entrapped in the PLGA nanoparticles via a modified multiple emulsion solvent evaporation method. The NPs displayed an increased DOX release at pH 5.5 compared to pH 7.4; the targeted NPs enhanced cellular uptake of DOX in C6 glioma cells, exerting a higher cytotoxic effect when compared with DOX solution alone [387]. Taken together, TNPs may lead to an improved therapeutic efficacy over DOX solution in glioma tumor growth inhibition for therapeutic and diagnostic purposes. Considering a similar strategy, chitosan NPs used as a dual action carrier for DOX (as chemotherapeutic agent) and superparamagnetic iron oxide nanoparticles (SPIONs; as imaging agent) were developed [388]. The TNPs demonstrated a pH-sensitive drug release profile with a burst release at acid tumor environment. TNP-DOX were internalized and the in vitro magnetic resonance imaging (MRI) showed a decline in T2 relaxation times by increasing iron concentration. The imaging method also confirmed uptake of TNPs at the optimum concentration in C6 glioma cells. Gold nanoparticles (AuNPs) and superparamagnetic iron oxide nanoparticles (SPIONs) were combined in a unique NPs to deliver therapeutic and diagnostic agents for brain tumors. The potential applications of novel gold and SPION-loaded micelles (GSMs) coated by polyethylene glycol-polycaprolactone (PEG-PCL) polymer was also tested [385]. The results showed, by quantifying gh2ax DNA damage in GB cell lines, the radiosensitizing efficacy of these GSMs, and found that GSM administration in conjunction with radiation therapy led to ~2-fold increase in density of double-stranded DNA breaks. GSMs used as a contrast agent for the MRI studies were sensitive to detect and delineate tumor borders. These results indicated that GSMs may potentially be integrated into both imaging and treatment of brain tumors, helping a theranostic purpose as both an MRI-based contrast agent and a radiosensitizer. Hollow gold nanospheres (HAuNS) have demonstrated an intense photoacoustic signal and induced an efficient photothermal ablation (PTA) therapy [389]. According to the in vivo results, these hybrid NPs significantly prolonged the survival of tumor-bearing mice. The results revealed the feasibility of the NP image-guided local tumor PTA therapy using photoacoustic molecular imaging. The application of multitargeting in a single NPs has also been described, and some examples are mentioned. Targeting AuNPs with two or more receptor binding peptides for glioblastoma treatment have been developed [251]. AuNPs conjugated with peptides against both the epidermal growth factor and transferrin receptors, and also loaded with the photosensitizer phthalocyanine 4 (Pc 4) was studied and compared with AuNPs employing in vitro and in vivo studies. The dual-targeting hybrid nanoparticles displayed a synergistic effect in human glioma cells and showed a significant accumulation in the brain tumor regions in in vitro studies and in vivo studies, respectively. 

The ability to perform multiple functions in biological systems, toxicity and biodistribution are the main requirements for the clinical translation of nanotheranostic concept. In general, the regulatory approval of NPs is challenging, due to their higher complexity as NDS. When TNPs, as multifunctional nanocarriers, are considered, the approval process has shown to be even more complicated, involving therapeutic, diagnostic and targeting agent assessments.

### 4.1. Personalized Medicine

Personalized medicine considers the pharmacogenetics, pharmacogenomics and pharmacoproteomic information as crucial for the prescription of therapeutics directed at each patient [390]. Thus, this new field considers the variability of each individual before developing an appropriate treatment strategy. The application of personalized medicine in the oncology field displays a promising strategy, due to the better understanding of the disease at a molecular level. Personalized medicine may be effective in the treatment and/or control of cancers with the implementation of a high specificity and accuracy of diagnosis [391]. Thus, the optimization of the treatment protocols becomes simpler and less expensive, associated with fewer toxicities and a high efficacy. Personalized cancer medicine (PCM) is defined as treatment based on the molecular characteristics of a tumor from an individual patient [392]. Recent researches have clarified several cellular and molecular mechanisms of tumor development, growth and metastasis with the identification of new cancer specific molecular targets. Taking advantage of pharmacogenetic, pharmacogenomic, pharmacoproteomic strategies, the detailed genetic and molecular profile of each patient, personalized medicine can help tailoring the design of nanoparticles. Targeted nanoparticles can interact with specific molecular biomarkers, which determine the evolution of the disease and the response to treatments [393]. Nanoparticles used in the personalized medicine are optimized treatment to each patient, taking into account the individual variability. However, critical steps are important in the personalized cancer therapy development, including a comprehensive assessment of biological characteristics of tumors from each individual and validated methods to identify the groups or subgroups of patients with more benefits in that therapy. Personalized medicine applied to a cancer presents a good example of a therapy relying on the individual genotype. The main difficulty in the translation to the clinic of nanoparticles in the context of personalized medicine is that it requires a comprehensive and detailed understanding of the potential toxicological risk associated to their use. If this difficulty can be overcome, clinical application of nanoparticles would allow to detect variation among patients, thus driving the clinical decisions and the elaboration of therapeutic protocols.

**Scheme 2:**
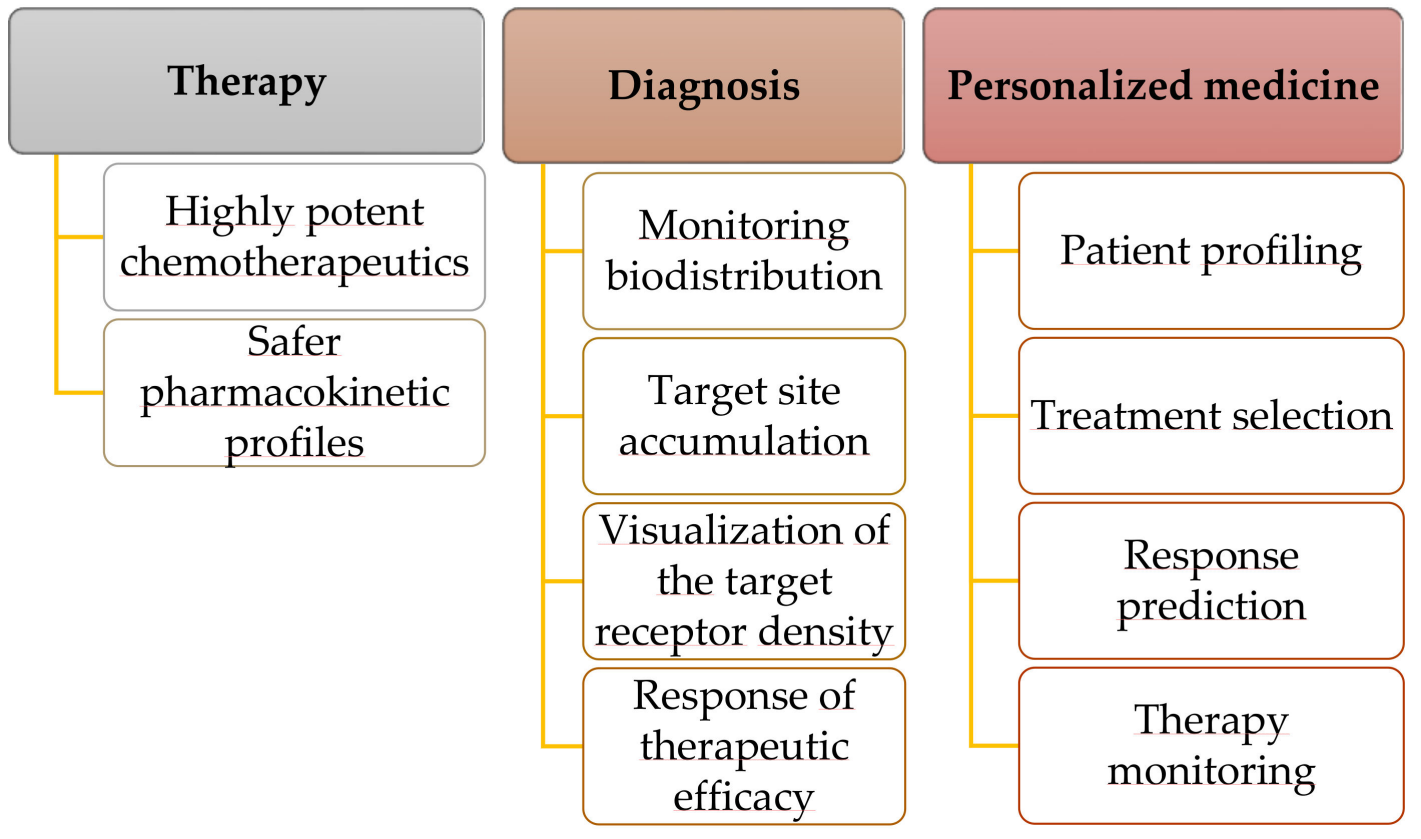
Personalized medicine strategy considering the use of theranostic nanoparticles.

As described in Section 3, the GB heterogeneity, their invasiveness and the numerous mutations, contribute to the progression of the tumor and the treatment failure [394,395,396]. Due to the different patient responses to conventional oncological treatments, the use of nanotechnology associated with personalized medicine is promising for the improvement of current strategies and the success of the treatment (Scheme 2). Targeting tumor heterogeneity and immune system may be applied using multifunctional nanoparticles, which may help prevent recurrence in GB by specific eradication of tumor cells. TNPs can permit the co-development, in single nanoparticles, of therapy, imaging and targeting approach, matching the most effective treatment to the individual patient. Scheme S1 sums up the aspects that need to be taken into account for this purpose.

Thus, it is predicted that personalized medicine along with the knowledge of GB environment with use of TNPs will improve their therapeutic and diagnosis efficacy. TNPs for personalized medicine will emerge and enter clinical trials. However, before this becomes a reality, safety concerns, problems related to the maintenance of robustness and reproducibility in the up-scaling processes must be addressed. 

### 4.2. Preclinical Phase and Clinical Trials

As it has been discussed, NPs are promising for applications in cancer theranostics because of their affinity and selectivity to tumor cells. In addition, they can simply be surface modified to increase their blood circulation times and make them functionalized with active targeting ligands. Recent advances in TNPs research have resulted in a number of formulations containing both drugs and imaging agents within a single formulation. However, despite the progress in the TNPs, their translation to the market has been a challenge. There are some researches focused on the preclinical trial phases, studying essentially the toxicity, pharmacodynamics, and safety profile of the TNPs. The main studies occur in vitro and in vivo, establishing the knowledge about the effect of a wide range of NPs doses to be tested, using cancer cell lines versus normal cell lines, and, as in a second step, in vivo assessment with appropriate animal models. Here, it is possible to evaluate the biocompatibility and the behavior of NPs with direct relation with the animal. The main purpose of the preclinical trials is to move forward the TNPs from the preliminary stage to the clinical phase.

Theranostic NPs, that combined organic nanoparticles (including liposomes, micelles/polymeric micelles, lipid nanoparticles, protein based nanoparticles) with co-delivery of drugs, and inorganic NPs (e.g., QDs, gold nanoparticles, and silica nanoparticles) as imaging contrast, have been developed and the major part of the results displayed a higher effectiveness than the commercial references [397,398,399,400,401,402,403,404,405]. The explosion of NPs for clinical imaging and therapy has not occurred yet. Their higher complexity and dubious reproducibility will create significant hurdles for clinical translation and regulatory approval. Although there are described several organic NPs for treatment and inorganic NPs for the imaging application approved or in clinical trials, these approaches did not represent a theranostic approach. INFeD^®^, DexIron^®^/Dexferrum Feridex^®^, Feraheme^TM^ (Ferumoxytol), NanoTherm^®^, Venofer^®^, AuroLase Therapy and AuNPs-nanosensor are some inorganic nanoparticles in current clinical trials or approved. Only NanoTherm^®^ is an intratumoral thermotherapy used for GB treatment, and the treatment is based on the principle of introducing magnetic nanoparticles directly into the tumor and then heating it using an alternating magnetic field [406]. A minimally invasive surgical procedure is used to introduce the magnetic fluid into the tumor followed by placing of the thermometry catheter in the treatment area to allow direct measurement of the temperature during thermotherapy. Although there are several TNPs under development, most of them do not yet possess a preclinical proof of concept, due to failure in the correlation between the in vitro and the in vivo results.

## 5. Conclusions

GB is one of the most dismal and mortal diseases, with no effective treatment. The BBB and BBTB, as the main physiological barriers, heterogeneity and the invasive nature of GB, lead to an inadequate concentration of chemotherapeutics at the site of tumor, restricting current treatments. These difficulties can be overcome using nanotechnology, which has demonstrated to be an opportunity in the area of cancer treatment. Thus, the NPs utilization may lead to a breakthrough in brain cancer management, due to their small size, high surface functionalization and, more recently, the opportunity to co-develop treatment and imaging in a single NP. In this review, it is shown that optimization of NPs in theranostics is a multivariable, multi-objective task that does not allow to point in a single direction. Theranostic NPs are smart carriers, able to diagnose, deliver and monitor the therapeutic response in real-time. Targeting overexpressed proteins and receptors on brain cancer cells allows a specific release of cargo in the exact site. The knowledge about the chemotherapeutics concentration and safer pharmacokinetic profiles, with a real-time monitoring of biodistribution and target site accumulation, visualization of the target receptor density and assessment of the therapeutic efficacy may be beneficial in the selection of therapy and planning the treatment. In the coming years, the global perspective for the use of TNPs is clearly optimistic, in multifunctional applications and in combination with personalized medicine strategies, offering hope for their successful clinical translation. Providing treatment at the right time depends on right-time diagnosis. Personalized cancer planning, advance diagnosis, and suitable drugs for the right patient, with predictable side effects, can finally make this goal a reality. Modern multimodality treatment and care increase patient’s life quality and life expectancy. It is probable that, in the next years, TNPs will emerge and enter clinical trials.

## Figures and Tables

**Table 1:** Current glioblastoma (GB) treatments: limitations and advantages.

Current Treatments	Pros	Cons	References
**Surgical resection**	Significant increase in survival rate. Possibility to apply radio-and chemotherapy, in order to remove residual tumor cells.	Damage of the surrounding cortex or brainstem structures, due to the diffuse nature of the tumor and inability to remove it.	[5]
**Radiotherapy**	Most frequent treatment.	Tumor response depends on its size. Acute side effects, such as damage of the epithelial surface, mouth, throat, gastrointestinal ulcers, swelling and infertility. Late effects, including fibrosis, hair loss, lymphedema and heart diseases. Effect only on the periphery of the tumor, with the core still being able to proliferate.	[6]
**Chemotherapy**	Cytotoxic and cytostatic agents act in tumor cells death through multiple mechanisms: angiogenesis, pro-differentiation, growth factor pathways and inhibition of tumor invasion. (e.g., temozolomide)	Several side effects including nerve damage, nausea, hair loss, vomiting, infertility, diarrhea, insomnia and skin rash. Effect only on the periphery of the tumor, with the core still being able to proliferate.	[6]
**Hyperthermia**	Tumor cell eradication based on generation of heat at the target site. It induces physiological changes, which lead to their apoptosis. Temperature ranges from 41 °C to 46 °C, activating many intracellular and extracellular degradation mechanisms.	Late effects including problems associated with heart, blood vessels, and other major organs.	[6,7]
**Immunotherapy**	Promotion of an enhanced anti-tumor immune response with an adequate antigen presentation, and circumvention of immunosuppressive mechanisms. Immunotherapy may include:T-Cell based vaccine therapies: EGFRvIII vaccine, heat-shock protein (HSP) vaccine, dendritic cell (DC) vaccines, adoptive T-cell therapy. Immune Checkpoint Inhibition: Anti-PD1, anti-CTLA4. Adoptive T-Cell Therapy: chimeric antigen receptors (CARs) targeting proteins (IL-13 receptor, Her2, EphA2, and EGFRvIII.	Low response rates: only a relatively reduced fraction of patients obtain clinical benefit. Potential increase in the magnitude, frequency, and onset of side effects. Severe immunological reactions, including a systemic cytokine release syndrome (“cytokine storm”), cause a delayed and/or inappropriate response, and may contribute to tissue damage.	[6,8,9,10,11,12,13]
**Gene Therapy**	Direct inhibition of the expression of oncogenes and normalization of tumor suppressor gene expression. Gene therapy include: Suicide genes: HSV-TK, CDA, carboxypeptidase G2 and CYP450. Immunomodulatory genes: IFN-beta, IL-4, -12, -18, -23. Oncolytic virotherapy: Herpes simplex virus, CR adenovirus, measles virus.Tumor-suppressor genes: p53, p16, p27 and PTEN.	Deficiency of antigen presenting cells inside the brain. Inefficient distribution, resulting in a poor delivery of a gene to the tumor cells.	[14,15,16,17]

**Table 2:** Specific transporters expressed on BBB.

Type of Transport	Example Ligands	Biological Significance	Reference
**Glucose receptors (GLUT)**	Mannose; Glucose	GLT1 targeting occurs when the NPs are coated with mannose	[47,48]
**Monocarboxylate transporter (MCT)**	Lactate; Short-chain fatty acids; Biotin; Salicylic acid; Valproic acid; Phenylbutyrate; 3,5,3’-triiodo-l-thyronine	MCT inhibitors: MCT1 and MCT2 would play a role in tumor maintenance; MCT4 would increase tumor aggressiveness	[49]
**Neutral amino acids transporter (NAAT)**	Tyrosine; Thyroid hormones (e.g., triiodothyronine); Asparagine; Histidine; Isoleucine; Leucine; Methionine; Phenylalanine; Threonine; Tryptophan	Transportation of neutral amino acids and some drugs, such as L-dopa and anticonvulsant gabapentin	[50,51,52,53]
**Cationic amino acids transporter (CAATs)**	Arginine; Lysine		[43]
**Anionic amino acids transporters (AAATs)**	l-glutamate; l-aspartate		[44,54]
**ßeta amino acid transporter (ßAATs)**	Beta (β)-alanine		[44]
**Choline transporter (ChT)**	Choline; Thiamine	Support of the neurological supplies of brain	[45,46]
**Peptide transporters (PT)**	Oligopeptide transporters (e.g., PepT1, PepT2); Polypeptide transport system (e.g., Oatp2, OAT-K1, OATP)	Covalently linked to a vehicle: chlorotoxin-based strategies	
**Fatty acid transporters (FAT)**	Fatty acids	Glioma cells use fatty acids as a substrate for energy production. Targeting lipid metabolism is a promising approach in treating malignant gliomas Energetic role: stimulate numerous neural functions, or performing potential pharmacological targeting	[55,56,57,58]
**Nucleoside transporters (NTs)**	Nucleoside transporters (ENT1, ENT2, ENT3, and ENT4); Concentration nucleoside transporters (CNT2)	NTs act as second messengers in many signal transduction pathways, in maintaining the homeostasis of the nucleosides within the CNS, such as adenosine, to keep it available to bind to receptors Important for recycling pathways for nucleosides transportation into CNS tissue that brain cannot synthesize	[59]

**Table 3:** Cell penetrating peptides described in the literature for the application on glioblastoma treatment.

CPPs	Sequence	Mechanism of Action	Type of Interaction	TTP	References
**Transactivating-transduction (TAT)**	AYGRKKRRQRRR	Endocytosis Micropinocytosis Pore formation		RGD	[106,107,108,109]
**R_8_ (cell penetrating peptide octa-arginine)**	RRRRRRRR		Affinity to neuropilin-1 (NRP-1)	RGD	[110]
**IL-13p (Interleukin 13 peptide)**	TAMRAVDKLLLHLKKLFREGQFNRNFESIIICRDRT		Affinity to IL13Rα2 receptor		[111]
**LIMK2 NoLS (nucleolar translocation signal (NoLS) sequence of the LIM Kinase 2 (LIMK2))**	KKRT LRKN DRKK RC				[112,113]
**Leptin30**	C-terminal (YQQVLTSLPSQNVLQIANDLENLRDLLHLLC)	transcytosis across the BBBmediated endocytosis pathway.			[114]
**peptide1-NS∆**	TCTWLKYH	(unknown)			[100]
**_D_(KLAKLAK)_2_**		Disruption of the mitochondria membrane		NGR CGKRK	[115]
**pVec**	LLIILRRRIRKQAHAHSK-NH_2_	non-endocytic pathway		gHoPe2	[104]
**Penetratin**	CKRRMKWKK	Direct penetration, endocytosis			[116,117]
**NFL-TBS**		Direct penetration, endocytosis			[118]
**SynB1** **SynB3**	RGGRLSYSRRRFSTSTGR RRLSYSRRRF	Direct penetration, endocytosis		Conjugated with elastin-like polypeptide	[101,119]
**Transportan 10** **(TP10)**	AGYLLGKINLKALAALAKKIL	Membrane Potential		-	[120]
**D-Maurocalcine** **(D-MCa)**	Cys_3_−Cys_17_, Cys_10_−Cys_21_, and Cys_16_−Cys_32_ (Positive-charge)	Membrane Potential		-	[121]
**DK17**	DRQIKIWFQNRRMKWKK-NH_2_	Membrane Potential		-	[122]
**CB5005**	KLKLALALALAVQRKRQKLMP	Membrane Potential	blocking agent of the NF-kB pathway	-	[123]
**ACP**	R_8_-EEEEEEEE (E_8_)	Membrane Potential (R_8_)	MMP-responsive (E_8_)	Angiopep-2	[124]
**GPLGVRGDG**			MMP-responsive	RGD	[125]
**polyarginine peptides**	(NH_2_-RRRRGRRRRKGC)		MMP-responsive		[126]
**(CKRRMK^Nvoc^WK^Nvo0c^K^Nvoc^)**	Derived from CKRRMKWKK	cellular uptake after rapidly cleaving the photolabile-protective group.	photo-responsiveness		[98]
**LNP**	KKRT LRKN DRKK RC	nucleolar translocation signal	pH-responsive		[112]
**H7K(R2)2**	RRK(HHHHHHH)RR	cross cell membranes in a seemingly energy-independent manner	pH-responsive		[127,128]
**TH**	(AGYLLGHINLHHLAHL(Aib)HHIL-NH_2_	histidine-rich TH peptide	pH-responsive	RGD	[102,129,130]
**R_6_H_4_**	RRRRRRHHHH	Clathrin-mediated Caveolae/lipid raft-mediated	pH-responsive	HA	[131]

**Table 4:** Description of the morphology and permeability phases of the blood–brain–tumor barrier (BBTB).

GB Phases		Morphology BBTB	Permeability BBTB
**First phase**	Initial phase of malignant brain tumors	Brain capillaries provide enough nutrients for their growth. The capillaries are continuous and non-fenestrated.	BBTB integrity is not compromised
**Second phase**	Progression of the tumor	Cancer cells invade neighboring healthy cerebral tissues. High metabolic demands. Tumor volume increase. New capillaries have fenestrations (12 nm)	BBTB integrity is compromised, increasing the permeability and the molecules with size below 12 nm may pass through.
**Third phase**	Tumor growth	Inter-endothelial gaps are formed between cerebral endothelial cells Fenestration size increase from 48 nm to 1 µm.	BBTB damage and enhanced permeability and retention (EPR) effect favors NPs accumulation in the tumor tissues.

**Table 5:** List of actively targeted nanoparticles (NPs) to surface cell markers, signaling pathways and tumor microenvironment directed essentially at GB treatment.

Targeting	Molecules	References
**BBB**		
**Ecs; P32; Neuropin (NRP-1/2); Aminopeptidase N (CD13)**	Wheat germ agglutinin (WGA), Folic acid (FA), CVNHPAFACGYGHTMYYHHYQHHL-NH_2_, TNG; (TGNYKALHPHNG); LyP-1 (CGNKRTRGC); iNGR (CRNGRGPDC), L-peptide A7R (termed LA7R); NGR	[285,286,287,288,289,290,291,292]
**Glucose receptors (GLUT)**	2-deoxy-D-glucose; Mannose	[293,294]
**Transferrin receptor (Tfr)**	T7 (HAIYPRH); Angiopep-2; Transferrin (Tf_pep_); Melanotransferrin (MT); OX26 and RI7217	[64,65,66,251,295,296,297,298]
**Low density lipoprotein receptor (LDLR)**	Polypeptide LHRH-ELP-C8; Trichosanthin (TCS); Angiopep-2 (TFFYGGSRGKRNNFKTEEY); Lactoferrin (Lf); apolipoprotein E (ApoE); Polysorbate 80; polyoxyethylene sorbitol oleate (PSO)	[73,74,75,76,77,78,79,124,285,298,299,300]
**Insulin receptor (IR)**	Cixutumumab; GL56; 83-14 Mab; 29B4 Mab	[79,80,301,302]
**ABC (ATP-binding cassette)**	d-Alpha-tocopheryl polyethylene glycol 1000 succinate (TPGS); Polysorbate 80; Polyoxyethylene sorbitol oleate (PSO)	[77,78,79,80,299,303,304,305]
**TME**		
**Hypoxic response**	HIF-1α inhibitor; EZN-2968; Hsp90; CAIX inhibitor; LyP-1 (CGNKRTRGC)	[181,306,307]
**Acid pH**	TH peptide (AGYLLGHINLHHLAHL [Aib]HHIL-NH_2_); pHLIP (ACEQNPIYWARYADWLFTTPLLLLDLALLVDADET); R_6_H_4_; H_7_K(R_2_)_2_; LNP	[96,102,112,127,128,129,130,131,279]
**Enzymatic alterations**	R9; TAT; LMWP; ACP; GPLGVRGDG; polyarginine peptides	[124,125,126,280,281,282]
**Temperature**	L-zipper peptide; (VSSLESKVSSLESKVSKLESKKSKLESKVSKLESKVSSLESK); ELP (VPGXG)	[308,309,310]
**BBTB**		
**Integrins**	RGD; c(RGDyK); NGR	[146,150,286,311,312,313,314,315,316,317,318,319]
**13 receptorα2 (IL-13Rα2)**	Pep-1 (CGEMGWVRC)	[320,321]
**EGFR**	Cetuximab; Inhibitor of COX-2 (e.g., Celecoxib); 125I-mab 425; PKI166; Canertinib; Pelitinib Monoclonal antibody C225; _D_(KLAKLAK)_2_; Epidermal growth factor (egf_pep_)	[251,322,323,324,325]
**EGFR variant III mutant (EGFRVII)**	F25P preproinsulin; CAR-engineered NK cell lines such as NK-92; Rindopepimut; D2C7-IT; PEPvIII (H-Leu-Glu-Glu-Lys-Lys-Gln-Asn-Tyr-Val-Val-Thr-Asp-His-Cys-OH)	[326,327,328,329]
**VEGF-1, -2**	Vatalanib; Bevacizumab; Tivozanib; L-peptide A7R (termed LA7R); K237 (HTMYYHHYQHHL-NH_2_)	[287,330,331,332]
**ECM**		
**Matrix metalloproteinases (MMPs)**	inhibitor of matrix protease-3 (TIMP-3); histone deacetylase inhibitors; Chlorotoxin; LMWP	[253,333,334,335,336]
**Fibrin**	cysteineearginineeglutamic acidelysineealanine (CREKA)	[337,338]
**Tenascin-C**	FHK (FHKHKSPALSPV)	[143,339]
**Angiopoietin 2**	MEDI3617; AMG780; Nesvacumab (REGN910); CVX-060	[332,340]
**Cox-2**	COX-2 inhibitors: celecoxib; Alantolactone; Plumbagin	[341,342,343]
**NF-kB**	Baicalein (BA); DYT-40; SN50; Natural compounds: Curcumin, Resveratrol, 2-cyano-3,12-dioxooleana-1,9(11)-dien-28-oic acid (CDDO); Non-steroidal anti-inflammatory drugs: Irinotecan and celecoxib; Sulfasalazine, Disulfiram, Glutathione; Antibodies (bortezomib, lactacystin, and MG132)	[153,189,344,345,346,347]
**TAMS**		
**Cxcl12/cxcr4**	Plenrixafor (DY-[NMe]DOrn-R-2Nal-G); Peptide R (RACRFFC); NT21MP (LGASWHRPDKCCLGYQKRPLP); Nef-M1 (NAACAWLEAQ)	[167,348,349,350]
**Nrp-1/2**	LyP-1 (CGNKRTRGC); TT1 (CKRGARSTC); Plumbagin	[351]
**Multi-ligand endocytic receptor mannose receptor (CD206/MRC1)**	CSPGAKVRC	[352]
**Colony stimulating factor 1 (CSF1)**	Emactuzumab (RG7155); AMG820; IMC-CS4 (LY3022855); PLX7486	[353]
**CD11b+ F4/80high M2-like tams**	M2pep (YEQDPWGVKWWY)	[354,355]
**CSF-1r**	RG7155	[164]
**Tumor fibroblasts**		
**Fibroblast growth factor (FGF)**	brivanib, nindetanib, cediranib, lenvatinib, sulfatinib, dovitinib, ponatinib and lucitanib; SSR12819E; C2KG2R9	[356,357,358,359]
**Glioma Stem Cells, Invasion and Metastasis**		
**VEGFR**	D16F7	[360]
**CD44**	HA; Peptide 7 (FNLPLPSRPLLR)	[154,361]
**CD133**	GL1 (LLADTTHHRPWT); CBP4	[193,362]
**PI3k/Akt/mTor**	PP242, P30 and NVP-BEZ235; Temsirolimus; Sirolimus; Everolimus; XL765 (SAR245409)	[363,364]
**Transforming growth factor-beta (TGF-β)**	AP-12009; SD-208, SB-431542; PDX; LY2109761 and LY364947 (HTS466284)	[365,366,367]
**Platelet-derived growth factor (PDGF)**	MEDI-575; Tandutinib (MLN518); Crenolanib (CP-868-596)	[368,369,370]
**Hedgehog**	GL1 peptide (LLADTTHHRPWT); CVNHPAFAC-NH2; CK (CVNHPAFAC-HTMYYHHYQHHL)	[287,371,372]
**Mammary-derived growth inhibitor (MDGI)**	CooP (CGLSGLGVA)	[373]

## Supplementary Materials

The following are available online at http://www.mdpi.com/1999-4923/10/4/181/s1, Scheme S1: Opportunities and challenges in the optimization of theranostic NPs as a multivariable, multi-objective approach.
