# Supplementary material for: Targeted Theranostic Nanoparticles for Brain Tumor Treatment
Source: Pharmaceutics. 2018 Oct 9;10(4):181. doi: 10.3390/pharmaceutics10040181 (PMC6321593; doi:10.3390/pharmaceutics10040181)

pharmaceutics

MDPI

Supplementary Materials: Targeted Theranostic Nanoparticles for Brain Tumor Treatment

Maria Mendes, João José Sousa, Alberto Pais and Carla Vitorino

Scheme A1. Opportunities and challenges in the optimization of theranostic NPs as a multivariable, multi-objective approach.

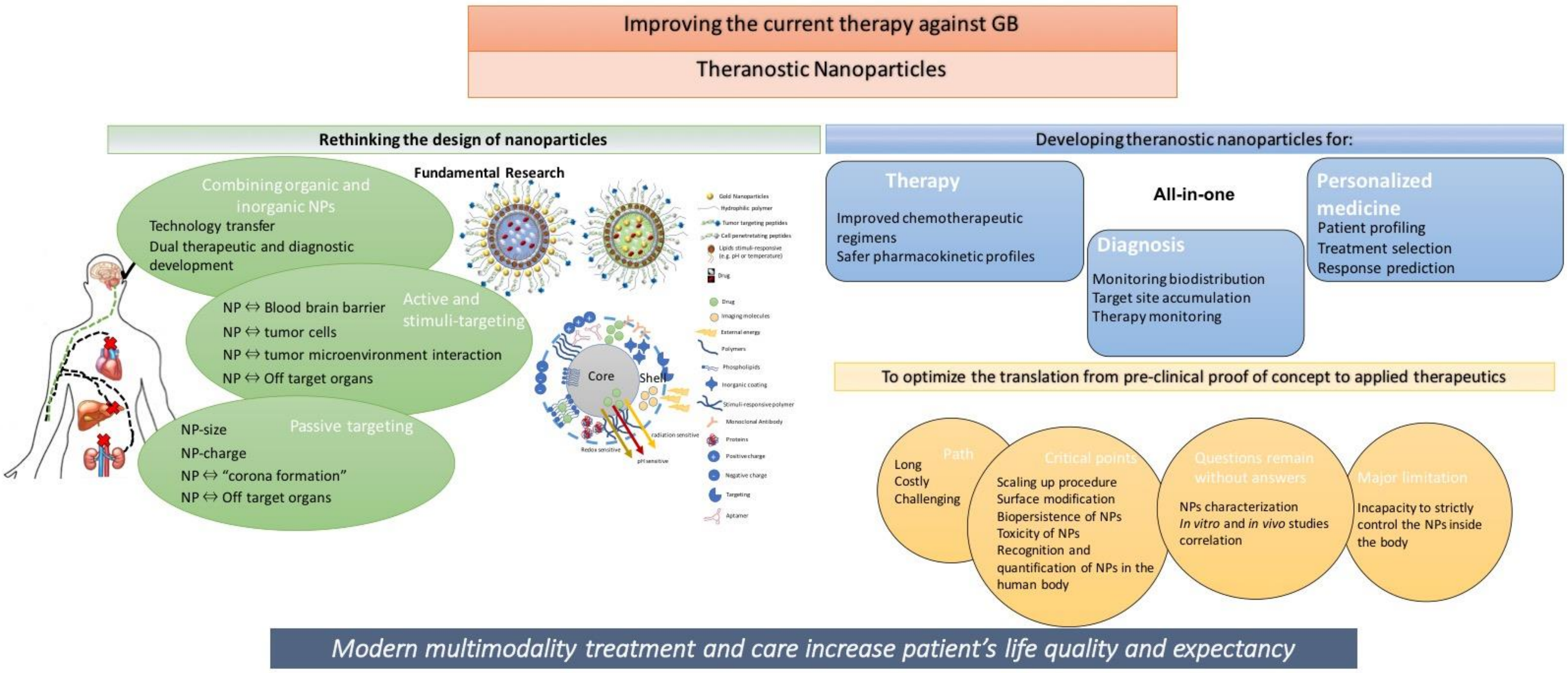

Supplement: Supplementary file 1 [file pharmaceutics-10-00181-s001.pdf]
